# The myth and reality of familial resemblance in dietary intake: a systematic review and meta-analysis on the resemblance of dietary intake among parent and offspring

**DOI:** 10.1016/j.eclinm.2023.102024

**Published:** 2023-06-02

**Authors:** Sonia Pervin, Pauline Emmett, Nick Townsend, Tuhin Biswas, M Mamun Huda, Kate Northstone, Yaqoot Fatima, H. David McIntyre, Abdullah Al Mamun

**Affiliations:** a Institute for Social Science Research, The University of Queensland, Brisbane, Australia; b ARC Centre of Excellence for Children and Families Over the Life Course, The University of Queensland, Brisbane, Australia; c Centre for Academic Child Health, Population Health Sciences, Bristol Medical School, University of Bristol, Canynge Hall, 39 Whatley Road, Clifton, Bristol, BS8 2PS, UK; d Centre for Exercise, Nutrition and Health Sciences, School for Policy Studies, University of Bristol, 8 Priory Rd, Bristol, S8 1TZ, UK; e Science and Math Program, Asian University for Women, Chattogram, Bangladesh; f Poche Centre for Indigenous Health, Faculty of Health and Behavioural Sciences, The University of Queensland, 74 High St, Toowong QLD 4066, Australia; g Population Health Sciences, Bristol Medical School, University of Bristol, Oakfield House, Oakfield Grove, Bristol, BS8 2BN, UK; h Centre for Rural and Remote Health, James Cook University, Mount Isa, Queensland, Australia; i Mater Clinical Unit and Mater Research, Faculty of Medicine, The University of Queensland, Raymond Terrace, South Brisbane, Queensland, 4101, Australia

**Keywords:** Parent-child pairs, Family, Dietary behaviours, Dietary resemblance, Whole diet

## Abstract

**Background:**

There is a strong societal belief that parents are role models for their child's dietary behaviours in early life that may persist throughout the life course. Evidence has shown inconclusive dietary resemblance in parent-child (PC) pairs. This systematic review and meta-analysis aimed to examine dietary resemblance between parent and children.

**Methods:**

We systematically searched for studies on PC dietary resemblance, via six electronic databases (PubMed, Ovid MEDLINE, Embase, APA PsycNet, CINAHL, and Web of Science) and other grey sources of literature between 1980 and 2020. We performed quality effect meta-analysis model on transformed correlation coefficients (z) to examine the resemblance in dietary intakes including nutrient intakes, food group intakes and whole diet. Finally, the Fisher's transformed coefficient (z) was used for meta-regression analysis to identify potential moderators. Heterogeneity and inconsistency were examined using the Q and I^2^ statistic. The study is registered on PROSPERO, CRD42019150741.

**Findings:**

A total of 61 studies met the inclusion criteria for systematic review, 45 were included in the meta-analysis. Pooled analyses showed weak to moderate PC dietary intake associations for energy: (r: 0.19; 95% CI: 0.16, 0.22), fat (% energy): (r: 0.23; 95% CI: 0.16, 0.29), protein (% energy): (r: 0.24; 95% CI: 0.20, 0.27), carbohydrate (% energy): (r: 0.24; 95% CI: 0.19, 0.29), fruits and vegetable (g/d): (r: 0.28; 95% CI: 0.25, 0.32), confectionary food (g/d): (r: 0.20; 95% CI: 0.17, 0.23), and whole diet (r: 0.35; 95% CI: 0.28, 0.42). Dietary intakes associations by study characteristics, including population, study year, dietary assessment method, person reporting dietary intake, quality of the study, and study design were highly variable, but associations were similar between PC pairs.

**Interpretation:**

The resemblance among parent-child pairs was weak to moderate for most aspects of dietary intakes. These findings challenge the social myth that parental dietary intake behaviour shapes their child's dietary intake.

**Funding:**

None.

**Research in context**

**Evidence before this study**

In a preliminary search of PubMed, MEDLINE, Embase, APA PsycNet, CINAHL, Web of Science database, Google, Google Scholar and ISRCTN registry, we scoped the existing evidence on the parent-child resemblance in dietary intake between January 1980 and December 2020, with no restriction by language. Our search terms included “association OR resemblance OR similarity OR concordance AND “familial OR parent∗ OR father∗ OR mother OR child OR children OR offspring” AND “food habit OR eating OR diet OR food intake OR dietary intake” and we did not limit the search by offspring age. We identified a number of studies on resemblance in dietary intake of children, adolescent, and pre-adult children in relation to their parents. A systematic review and meta-analysis published in 2011 on the topic included 24 studies with 15 studies used in the meta-analysis. It had a limited number of dietary components including energy and fat intake, both absolute (in grams) and relative intake (% of energy derived from fat).

**Added value of this study**

This study used a comprehensive search including elaborated key words, several additional dietary components: nutrients (protein, carbohydrates); food groups (fruits and vegetables, confectionary foods) and the whole diet (overall consumption of foods); and added further peer-reviewed publications from 2010 to 2020. This updated systematic review was based on 61 relevant studies. From 61 eligible studies, 45 with 214 data points were used in the meta-analysis. Thus, the additional number of data points increasing the statistical power and ability to test the differences across sample characteristics compared to the earlier review. We found that parent-child resemblance in a various aspect of dietary intakes (nutrients and food groups) was consistently weak, whereas that with whole diet showed moderate associations. We also observed that dietary intake resemblance among parent-child varied by study population, publication years, dietary assessment method, the person who reporting dietary intake, quality of the study, and design of study, but was similar between parent-child, mother-child and father-child pairs.

**Implications of all the available evidence**

Findings of this review demonstrate that most of the studies are from higher socio-economic countries. The family structure, social norms and parenting practices of economically advantaged countries are quite distinct from those in low-economic regions and are likely to be driven by an individualized society perspective. It is likely that modern living encourages the practice of autonomous behaviours and decision-making from childhood, and this may influence the food choices of children which is independent of parental habits whether mother or father. Our findings challenge the social myth that parental dietary intake profoundly influences their child's dietary intake. The possibility that the dietary intake of children is subject to numerous social and environmental factors which are dissimilar from their parents should be explored. Further studies in middle- and low-income countries are needed. More research is required to establish the potential mechanism that explains the persistent weak to moderate association among parent and child dietary intakes.

## Introduction

Dietary behaviours (referring to the overall phenomena related to food choice, eating behaviour, dietary patterns and dietary intake or nutrition)^1^ are established in early life and may track throughout the life course.^2^ Early childhood is a critical period for the development of dietary behaviour. Healthy eating behaviours can reduce the risk of non-communicable diseases (NCDs) including diabetes, heart diseases, stroke, and cancer.^3^, ^4^, ^5^, ^6^, ^7^, ^8^ By contrast, unhealthy dietary behaviours^4^^,^^9^ are known to increase the risk of obesity and NCDs and various atopic disorders.^10^, ^11^, ^12^, ^13^ Therefore, it is essential to establish healthy eating behaviours in early childhood.

Children's dietary behaviours across different socio-cultural backgrounds may be influenced by their family members including parents/grandparents, caregivers, and siblings. In early childhood, parents and caregivers are responsible for food provision and can shape dietary intakes, including the type of foods eaten, the amounts consumed, and the timing of meals.^14^ For younger children, parents and caregivers, including grandparents or educators in childcare settings usually control dietary intake inside and outside of the home,^15^^,^^16^ and they can act as role models regarding healthy or unhealthy eating behaviours. Parents' and caregivers' own beliefs, attitudes, and practices regarding food can also influence dietary behaviour in children.^16^ However, a growing body of evidence suggests that parent-child resemblance in dietary intake may vary in different setting.^15^^,^^17^^,^^18^

For older children, dietary intake may be further influenced by children's peer groups and school environment. Thus, some modifications at this stage are more likely.^19^ However, one study has reported a stronger resemblance between parents and their older children (10–18 years) than with their younger children (2–10 years) in total and saturated fat intakes.^15^ Conversely, a weak dietary resemblance was found in a study of mothers with their young adult offspring (18–23 years), though, correlations tended to be stronger if offspring still lived with their parents.^18^ Furthermore, it is reported that shared meals in the family can promote resemblance in dietary intakes among family members.^20^^,^^21^ Modern changes in food environments, especially increased availability of foods that are poor in nutrient content (e.g., processed foods) and rapid transitioning from traditional food habit to modern food choice has led to changes in individual and familial dietary intakes.^22^, ^23^, ^24^, ^25^, ^26^

Globally, modernized busy lifestyles have transformed the traditional family model resulting in parents having less time to shop for and prepare healthy foods and less time for meals with the family.^27^ As a result, children spend more time away from the family, either at school or with caregivers and are more likely to be provided with ready prepared foods.^24^^,^^28^, ^29^, ^30^, ^31^ These changes are likely to have a profound effect on children's eating habits. An earlier systematic review and meta-analysis of the parent-child resemblance of dietary intake by Wang et al. (2011) summarized the overall association as low to moderate.^32^ The review explore associations for total energy, total fat and fat as a percentage of total energy and found that the relationship varied widely by studies characteristics including country, dietary assessment method used in studies, and the type of parent-child dyad.^32^ However, some studies suggested that parent-child dietary resemblance may be more robust for other nutrients or food items.^15^^,^^33^, ^34^, ^35^ Another systematic review by Yee et al. (2017) demonstrated parental role modelling as strong correlates compared to other parental practices. However, the overall effect sizes for all parental practice indicators were small to medium.^36^

Given the small number of studies identified in the previous review and meta-analysis, we anticipated an increase in the number of studies published since 2009, examining the diverse dietary intake components, along with different settings and age groups. There was a need to update the previous systematic review and investigate the resemblance between parent-child diet using additional dietary intake components. Therefore, our study aimed to extend the review of Wang et al. (2011) and to perform a new meta-analysis on the resemblance of dietary intakes between parent and offspring. This study included additional nutrients (protein and carbohydrates), food items (fruits and vegetables; as well as confectionary foods) and measures of the whole diet (the overall food consumption of an individual) using studies published from 1980 to 2020.

## Methods

We followed the Preferred Reporting Items for Systematic reviews and Meta-Analyses (PRISMA) 2020 guidelines^37^ for undertaking and reporting the systematic review and meta-analysis. The systematic review and meta-analysis protocol was registered in PROSPERO (CRD42019150741). This paper reports findings relating to the first domain (diet) of the registered objectives to quantify the parent-offspring resemblance in cardio-metabolic risk behaviours (diet, physical activity, sedentary behaviours, smoking and alcohol intake) and examine the contributing factors. In order to reduce the complexity of interpretation of the results, findings for other domains of the objectives will be reported separately.

### Search strategy and selection criteria

We performed a literature search on PubMed, MEDLINE, Embase, APA PsycNet, CINAHL, Web of Science database, Google, Google Scholar and ISRCTN registry from 1 January 1980 to 31 December 2020. The full search strategy is available in the Supplementary Table S1 (appendix pp 4–5). Studies that reported associations in dietary intake between parent and offspring without any age restriction were eligible for inclusion. We included parental dietary intake paired with child dietary intake to assess familial resemblance. Studies reporting only parents' knowledge, attitude, perception, parental/family support, parental behaviours, and family environment and association with their offspring's dietary behaviours were not eligible for inclusion.

We pooled all reported estimates of dietary intake associations from included studies. We identified nine different mutually exclusive categories of parent-child dyads according to the sex of parent and child, including parent-child (PC), parent-son (PS), parent-daughter (PD), father-child (FC), father-son (FS), father-daughter (FD), mother-child (MC), mother-son (MS), and mother-daughter (MD).^32^ We reported dietary intake variables for: overall energy (in kcal), relative intakes of fat, protein, and carbohydrate (% of total energy derived from these nutrients), two food groups: fruits and vegetables (g/day); confectionery foods (g/day) defined as confectionery, sugary snacks, sugar-sweetened beverages, candies and chocolates, and the whole diet. In this study, the term ‘whole diet’ refers to the consumption of all food items and beverages that provide energy and nutrients and constitute an individual's complete dietary intake. Whole diet was measured through variety of dietary assessment methods such as food frequency questionnaire (FFQ), 24-h recalls, food records. Diverse analytical methods were used to assess whole diet including dietary pattern analysis and diet quality indices.

Two independent reviewers (SP and TB) assessed all titles, abstracts, and full-text articles against the eligibility criteria. Any disagreement was resolved by discussion and a third reviewer (AAM) was involved where necessary. Two reviewers (SP and TB) independently scored the methodological quality of included studies using the Quality Assessment Tool for Observational Cohort and Cross-Sectional Studies developed by the National Institute of Health (NIH).^38^ The quality assessment tool contains 14 questions for quality assessment based on their relevance to observational studies is presented in the Supplementary Table S2 (appendix pp 6–9). The studies were, then classified in relation to the highest score as poor quality (if scored <0.60), fair quality (scored ≥0.60 to ≤0.80) and good quality (scored >0.80 to 1.00).

### Statistical analysis

We extracted the number of data points (e.g., correlation coefficients) based on dietary intakes of all PC pairs and study characteristics in the systematic review and meta-analysis. We used descriptive analysis to summarize data points by study characteristics using 214 data points from the 45 studies presented as counts and percentages. We presented descriptive statistics (as mean and 95% of confidence intervals (CIs)) on reported correlation coefficients (r) for each of the dietary intake variables. Then, reported effect measures were transformed from Pearson's or Spearman's correlation coefficients (r) by Fisher's z transformation (z's) to derive approximate normality and calculated the mean with 95% CIs.^32^ Further, we compared the difference in transformed correlation coefficients (z) for each of the dietary intake variables by different study characteristics using one-way ANOVA.^39^^,^^40^

We conducted a meta-analysis based on transformed z for each dietary intake variables, calculated pooled estimates with 95% CIs of r using MetaXL version 5.3^41^ and presented these in forest plots for parent-child, mother-child and father-child pairs. We used a quality-effects (QE) meta-analysis model incorporating a restricted maximum-likelihood variance estimator.^42^ In brief, the QE model is a bias adjustment method that computes synthetic bias from quality score that favours studies with better methodological quality.^43^ Using the QE model, the software computes the standard error of the logit-transformed coefficient with confidence intervals and variance for each study. Statistical heterogeneity and inconsistency was checked using the Q and I^2^ statistics, respectively.^44^ We performed subgroup analysis by PC pairs for extreme levels of heterogeneity between studies (I^2^ ≥ 90%). Sensitivity analyses were performed by adding random effect model and excluding poor quality of studies to assess the difference in pooled estimation.

We also assessed publication bias using both a graphical (Doi plot) and quantitative [Luis Furuya-Kanamori (LFK) index] examination for potential small-study effects^45^ (Supplementary Figure S1, appendix pp 26). The Doi plot visualises asymmetry by using a rank-based precision measure (Z score rather than SE) and plotting it against effect size. As part of the LFK index, which is calculated by averaging half of the sum of the Z score and the normalised effect size across the meta-analysis, which allows to identify and quantify the asymmetry of the Doi plots. The publication bias is not evident in the case of a symmetric shape, whereas an asymmetric shape indicates publication bias. The value of LFK index within −1 and +1 indicates no publication bias, an LFK of −1 to −2 or +1 to +2 are deemed consistent with minor asymmetry, and an LFK of <−2 or >+2 represents major asymmetry.

Finally, we fitted multivariable meta-regression analysis using quality effect models on transformed coefficient (z) to predict the possible factors associated with dietary resemblance across study characteristics. Several predictors were used in the metaregression model, including population, publication year, dietary assessment methods, who reported the dietary data, study design, and study quality scores (model 1), adding age of the child (model 2), parent-child pair ignoring child's sex (model 3), and parent-child pairs considering child's sex (model 4). All study predictors and transformed pooled estimations produced in MetaXL were imported to STATA software version 17.0 with which all analyses were performed.^46^ Effect size interpretation used as weak negative or null (if r < 0.00), weak (if r > 0.00 to <0.30), moderate (r ≥ 0.30 to <0.50) and strong (r: ≥0.50) association.^32^ In this study, a p-value of <0.05 was considered significant for all statistical tests.

### Role of the funding source

This research was not externally funded. The corresponding author has full access to all the data in the study and has final responsibility for the decision of submission to the journal for publication.

## Results

**Fig. 1:**
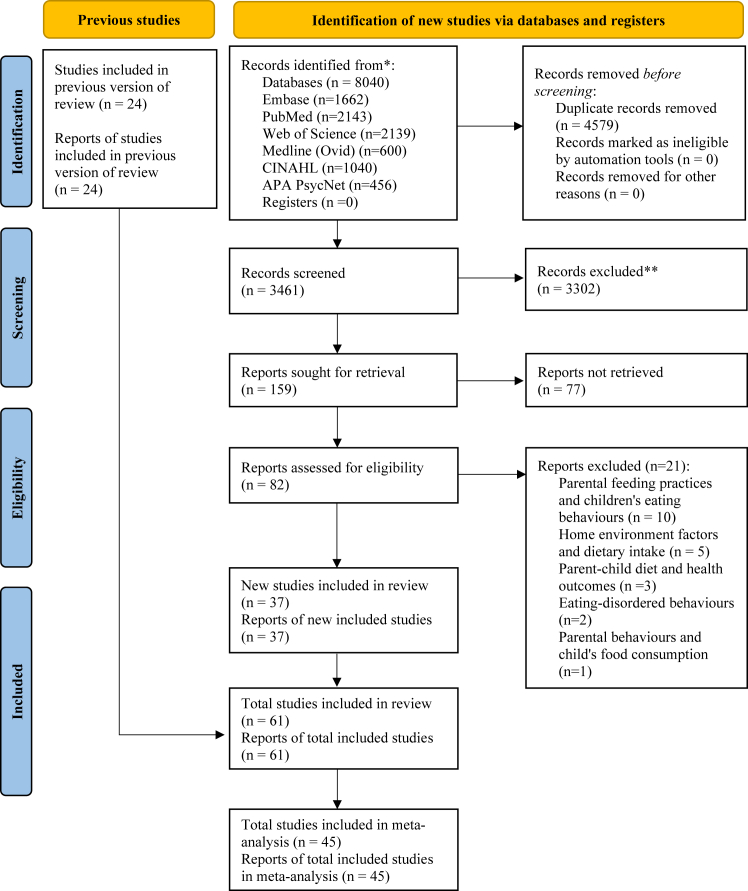
Data extraction flow chart. PRISMA 2020 flow diagram showing data extraction process of the study.

We identified 8040 published articles from the electronic database search. Details on study selection and reasons for exclusion are summarised in Fig. 1. Overall, a total of 61 studies met our inclusion criteria for systematic review, and 45 were included in a meta-analysis (Fig. 1). The main characteristics and findings of the 61 studies that reported parent-child resemblance in dietary intake are detailed in the supplementary appendix (Supplementary Table S3, appendix pp 10–27). The majority of the studies (58 [95.1%]) were in high income countries: USA (26 [42.6%]); European countries (21 [34.4%]); Australia (8 [13.1%]); Canada (1 [1.6%]); South Korea (1 [1.6%]); Japan (1 [1.6%]) and the rest (3 [4.9%]) were in lower- and middle-income countries. Most of the studies (49 [80.2%]) were published during 2000–2020. Over two-thirds (n = 47) were cross-sectional studies, whereas less than a third (n = 14) were longitudinal studies (Table 1). The sample size of the parent-child pairs varied from 36 to 4707.^47^^,^^48^

**Table 1:** PICOS criteria for inclusion and exclusion of studies.

Criteria	Description
Participants	Inclusion: Parent and their offspring. For parent, irrespective of biological and non-biological parents, either parent, at least one parent, both parents, offspring (children, adolescent, pre-adults, and adults)
	Exclusion: Studies solely among parents/offspring with a pre-existing health condition or studies solely among parents/offspring addressed as special population (mental health issues or disability).
Intervention/exposure	Inclusion: Association of parental dietary intake with their child's diet (self-reported or objectively measured before or at the same time as their offspring, once or multiple times). Parental and offspring dietary intake is assessed as: Macronutrient intake in grams: carbohydrates, protein, total fat, fatty acids (saturated and unsaturated). Micronutrient intake in grams: Vitamins and minerals. Dietary pattern/Diet scores/indices of diet quality. Food groups/consumption of food products (e.g., fruit intake, vegetable intake, soft drinks, dairy products, meat, sweets, and confectionary foods) mentioned in grams or standard portions.
	Exclusion: Studies examining parental perception of diet, knowledge and attitude regarding dietary intake and correlation with their child dietary intake.
Comparison	Inclusion: Not applicable in this study.
	Exclusion: Not applicable in this study.
Outcome	Inclusion: Offspring/child's dietary intake in relation to parental diet (self-reported or objectively measured before or after or at the same time as their parents, once or multiple times).
	Exclusion: Child dietary disorder
Study design	Inclusion: Cross-sectional, prospective observational or experimental studies
	Exclusion: Systematic review, reviews of systematic review.

Dietary intake was assessed using various methods: Food frequency questionnaire (23 [37.7%]), Food records (12 [19.7%]), 24-h recall (8 [13.1%]) the rest used multiple methods (9 [14.8%]) (Table 2). In over a third of studies (21 [34.4%]) the diets were self-completed (parent and child each reported diet for themselves); however, in mother reported (14 [23.0%]) studies, the mothers reported dietary intake for herself and her child. Twenty (20 [32.8%]) studies used both (self-completed and parent-reported intake for the child), and six 6 [9.8%]) studies collected dietary intake information using interviewers. Data on resemblance varied by PC pair and dietary intake variables. Out of 214 data points, reported PC resemblance of dietary intakes were most often weakly associated (147 [68.7%] data points), with some showing moderate associations (50 [23.4%] data points); weak negative associations (8 [3.7%] data points) or strong associations (9 [4.2%] data points) (Table 2). Of five studies (18 data points) that examined PC resemblance using whole diet intake, more than one-third (7 [38.9%] data points) had reported a strong association.

**Table 2:** Summary of the characteristics of studies and number of data points (i.e., correlation coefficients)^a^ for the resemblance of dietary intakes between parent and child.

Indicators	Type	Studies included in the review^b^	Studies included in the meta-analysis^c^	Data points from 45 studies included in the sub-group and meta-regression analysis^d^						
				Nutrient intake				Food intake		Whole diet
				Energy (kcal)	Fat (% kcal)	Protein (% kcal)	Carb (% kcal)	Fruits/Vegetables (g/d)	Confectionery foods (g/d)^l^	
		n (%)	n (%)	n (%)	n (%)	n (%)	n (%)	n (%)	n (%)	n (%)
**Total**		**61 (100)**	**45 (100)**	**57 (100)**	**30 (100)**	**32 (100)**	**19 (100)**	**28 (100)**	**30 (100)**	**18 (100)**
Populations^e^	USA	26 (42.6)	21 (46.7)	30 (52.6)	9 (30.0)	12 (37.5)	6 (31.6)	14 (50.0)	10 (33.3)	10 (55.6)
	European countries	21 (34.4)	15 (33.3)	16 (28.1)	17 (56.6)	10 (31.2)	10 (52.6)	9 (32.1)	13 (43.4)	6 (33.3)
	Australia	8 (13.1)	5 (11.1)	5 (8.8)	2 (6.7)	9 (28.1)	2 (10.5)	5 (17.9)	7 (23.3)	2 (11.1)
	Others∗	6 (9.9)	4 (8.9)	6 (10.5)	2 (6.7)	1 (3.1)	1 (5.3)	–	–	–
Publication year	1980s	3 (4.9)	3 (6.7)	15 (26.3)	–	–	1 (5.3)	–	–	–
	1990s	9 (14.7)	8 (17.8)	19 (32.3)	12 (40.0)	15 (46.9)	12 (63.1)	1 (3.6)	1 (3.3)	–
	2000s	22 (36.0)	16 (35.6)	13 (22.8)	11 (36.7)	1 (3.1)	–	11 (39.3)	13 (43.4)	5 (27.8)
	≥2010s	27 (44.2)	18 (40.0)	10 (17.6)	7 (23.3)	16 (50.0)	6 (31.6)	16 (57.1)	16 (53.3)	13 (72.2)
Dietary assessment^f^	FFQ	23 (37.7)	19 (42.2)	14 (24.5)	10 (33.3)	5 (15.6)	2 (10.5)	6 (21.4)	10 (33.3)	8 (44.4)
	24 h recall	8 (13.1)	6 (13.3)	16 (28.1)	10 (33.3)	5 (15.6)	4 (21.1)	11 (39.3)	11 (36.7)	10 (55.6)
	Food record	12 (19.7)	10 (22.2)	17 (29.8)	10 (33.3)	19 (59.4)	13 (68.4)	6 (21.4)	7 (23.4)	–
	Mixed	9 (14.8)	7 (15.6)	9 (15.8)	–	3 (9.4)	–	4 (14.3)	1 (3.3)	–
	Others∗	9 (14.8)	3 (6.7)	1 (1.8)	–	–	–	1 (3.6)	1 (3.3)	–
Diet data reported by^g^	Self-completed∗	21 (34.4)	17 (37.8)	15 (26.3)	6 (20.0)	7 (21.9)	1 (5.3)	10 (35.7)	10 (33.3)	–
	Mother	14 (23.0)	10 (22.2)	8 (14.0)	–	3 (9.4)	–	2 (7.1)	4 (13.4)	1 (5.6)
	Mixed	20 (32.8)	13 (28.9)	33 (57.9)	23 (76.7)	21 (65.6)	18 (94.7)	15 (53.6)	15 (50.0)	11 (61.1)
	Interviewer	6 (9.8)	5 (11.1)	1 (1.8)	1 (3.3)	1 (3.1)	–	1 (3.6)	1 (3.3)	6 (33.3)
Reported diet^h^	Food groups	30 (49.2)	20 (44.4)	–	–	–	–	–	–	–
	Nutrients	15 (24.6)	13 (28.9)	–	–	–	–	–	–	–
	Whole diet	8 (13.1)	5 (11.1)	–	–	–	–	–	–	–
	Mixed∗	8 (13.1)	7 (15.6)	–	–	–	–	–	–	–
Study design	Cross-sectional	47 (77.1)	33 (73.3)	40 (70.2)	19 (63.3)	7 (21.9)	3 (15.8)	15 (53.6)	14 (46.7)	18 (100)
	Longitudinal	14 (22.9)	12 (26.7)	17 (29.8)	11 (36.7)	25 (78.1)	16 (84.2)	13 (46.4)	16 (53.3)	–
Child age^i^	Child age (mean)	10.6 ± 4.6	10.6 ± 4.6	11.8 ± 3.3	12.9 ± 3.2	11.9 ± 3.9	9.9 ± 3.2	11.1 ± 2.8	10.9 ± 2.1	7.8 ± 2.8
Child age group^j^	Childhood	29 (47.5)	23 (51.1)	22 (38.6)	6 (20.0)	8 (25.0)	2 (10.5)	16 (57.2)	19 (63.4)	9 (50.0)
	Adolescence and above	13 (21.3)	10 (22.2)	10 (17.5)	5 (16.7)	4 (12.5)	–	3 (10.7)	1 (3.3)	–
	Both∗	19 (31.2)	12 (26.7)	25 (43.9)	19 (63.3)	20 (62.5)	17 (89.5)	9 (32.1)	10 (33.3)	9 (50.0)
Strength of association^a^, ^k^	Weak negative association	–	8 (3.7)	5 (8.8)	3 (10.0)	–	–	–	–	–
	Weak association	–	147 (68.7)	45 (78.9)	17 (56.7)	24 (75.0)	13 (68.4)	16 (57.1)	25 (83.3)	7 (38.9)
	Moderate association	–	50 (23.4)	7 (12.3)	10 (33.3)	8 (25.0)	6 (31.6)	12 (42.9)	3 (10.0)	4 (22.2)
	Strong association	–	9 (4.2)	–	–	–	–	–	2 (6.7)	7 (38.9)
Parent-Child pairs^a^	Parent-Child (PC)	–	38 (17.8)	13 (22.8)	5 (16.7)	6 (18.7)	3 (15.8)	6 (21.4)	4 (13.3)	1 (5.6)
	Parent-Son (PS)	–	1 (0.5)	–	–	1 (3.1)	–	–	–	–
	Parent-Daughter (PD)	–	1 (0.5)	–	–	1 (3.1)	–	–	–	–
	Father-Child (FC)	–	25 (11.7)	8 (14.0)	3 (10.0)	4 (12.5)	3 (15.8)	2 (7.1)	3 (10.0)	2 (11.1)
	Father-Son (FS)	–	27 (12.6)	6 (10.5)	5 (16.7)	4 (12.5)	3 (15.8)	3 (10.7)	4 (13.3)	2 (11.1)
	Father-Daughter (FD)	–	27 (12.6)	6 (10.5)	5 (16.7)	4 (12.5)	3 (15.8)	3 (10.7)	4 (13.3)	2 (11.1)
	Mother-Child (MC)	–	29 (13.5)	7 (12.3)	2 (6.5)	2 (6.3)	1 (5.2)	5 (17.9)	5 (16.7)	7 (39.9)
	Mother-Son (MS)	–	32 (14.9)	8 (14.1)	5 (16.7)	5 (15.6)	3 (15.8)	4 (14.3)	5 (16.7)	2 (11.1)
	Mother-Daughter (MD)	–	34 (15.9)	9 (15.8)	5 (16.7)	5 (15.6)	3 (15.8)	5 (17.9)	5 (16.7)	2 (11.1)
Quality score^m^	Poor	18 (29.5)	12 (26.7)	12 (21.0)	9 (30.0)	4 (12.5)	1 (5.3)	8 (28.6)	7 (23.3)	13 (72.2)
	Fair	20 (32.8)	17 (37.8)	23 (40.4)	4 (13.3)	7 (21.9)	1 (5.3)	6 (21.4)	9 (30.0)	4 (22.2)
	Good	23 (37.7)	16 (35.5)	22 (38.6)	17 (56.7)	21 (65.6)	17 (89.4)	14 (50.0)	14 (46.7)	1 (5.6)

“–” denoted for no data points for the corresponding study characteristics.

a Values are number of data points (n) and the percentages (%) within each characteristic. A total of 45 studies with 214 datapoints from seven diet groups were used for data analysis.

b Number of studies which were included in the systematic review and the percentages (%) within each characteristic.

c Number of studies included in the meta-analysis and meta-regression analysis [n and percentages (%) within each characteristic].

d Number of data points (parent-child correlation coefficients) are available for each dietary intake variables.

e Populations: ∗Others, countries included are Canada (Quebec), Nigeria, South Korea, Brazil, Japan, and Mexico.

f Dietary assessment method: FFQ, Food frequency questionnaire; 24 h recall, 24 h recall methods; Food record, 2/3 days food record; Mixed, used any two assessment methods from above; short questionnaire e.g., Fat-and-fibre-related diet behaviour questionnaire (FFB) and pro-children questionnaire (Child).

g Dietary data reported by: ∗Self-completed, both mother and child reported and completed diet for themselves; Mother, Mother reported for both herself and child; Mixed, parent (self-completed) and child (interviewer administered); ∗Interviewer, interviewer administered for both.

h Reported diets: Carb, Carbohydrate; whole diet, various types of diet quality score; ∗mixed reported both food groups and nutrients and/or whole diet.

i Values are Mean ± SD within each dietary intake variables.

j Child age group: Childhood includes early childhood (age 2 to <6 years) and Middle childhood (age ≥6 years to ≤12 years); Adolescence and above includes early adolescence (age >12 years to ≤16 years) and late adolescence/adult (age >16 years to ≤25 years). ∗Both, children are included from both age groups (childhood to adolescence).

k Strength of association: Weak negative or null association (r: <0.00), Weak association (r: 0.00 to <0.30), Moderate association (r: ≥0.30 to <0.50), Strong association (r: ≥0.50).

l Confectionery food items included: confectionery, sugary snacks, sweetened beverages, candies, and chocolates.

m Quality score: Poor quality (0–9 points), fair quality (10–13 points) and high quality (14–17 points).

Dietary resemblance was most reported for PC pairs without specifying the sex of either parent or child (38 [17.8%] data points) followed by MD pairs (34 [15.9%] data points). Most studies reported only one of the three types of dietary intake variable: food items (30 [49.2%] studies), nutrient intakes (15 [24.6%] studies), whole diet (8 [13.1%] studies) and the rest (8 [13.1%]) reported a combination of these. The reported mean age group was 10.6 ± 4.6 for children. The majority of studies (29 [47.5%]) included a child from either early or middle childhood (age 2–12 years). Some (13 [21.3%]) focused on offspring from adolescence and upwards (age 12–≤ 25 years). Other studies covered both (19 [31.2%]) age groups. For the 61 studies, the quality scores ranged from 6 to 17 points (Supplementary Table S2, appendix pp 4–7). More than one-third of studies were scored as good quality (23 [37.7%] studies), and rest were classified as fair quality (20 [32.8%] studies), and poor quality (18 [29.5%] studies) (Table 2).

**Fig. 2:**
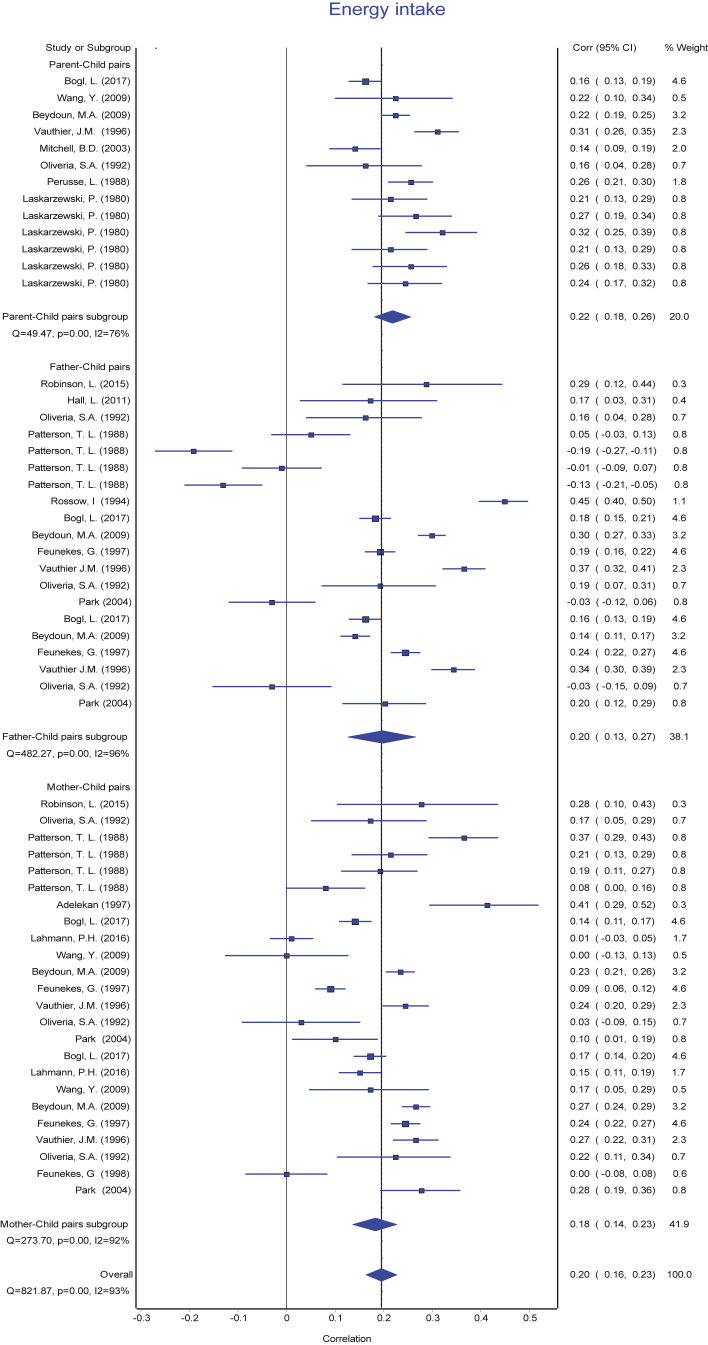
Forest plot of energy Intake. Comparing meta-analysis results of overall transformed correlation coefficient (z) on the association between parent and child resemblance on dietary intake in 45 studies for the three parents-child pairs. Pooled overall estimation with 57 data points for energy intake from 45 studies-r: 0.20 (95% CI: 0.16, 0.23); test for heterogeneity: Q = 821.87, I^2^ = 93%, p < 0.01.

**Fig. 3:**
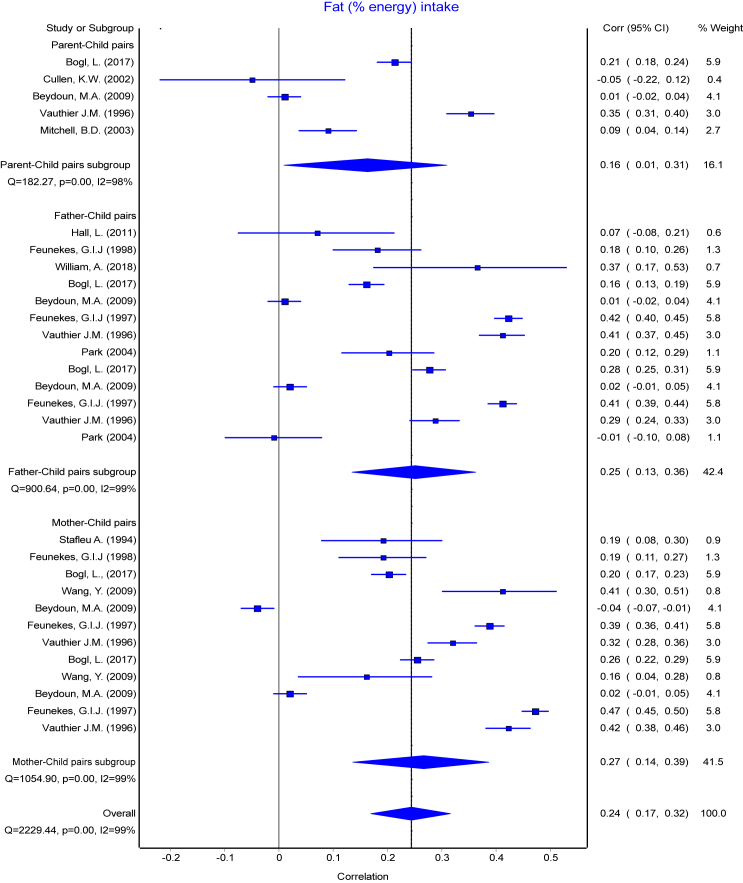
Forest plot of fat (% energy) intake. Comparing meta-analysis results of overall transformed correlation coefficient (z) on the association between parent and child resemblance on dietary intake in 45 studies for the three parents-child pairs. Pooled overall estimation with 30 data points for fat (% energy) intake from 45 studies-r: 0.24 (95% CI: 0.17, 0.32); test for heterogeneity: Q = 2229.44, I^2^ = 99%, p < 0.01.

**Fig. 4:**
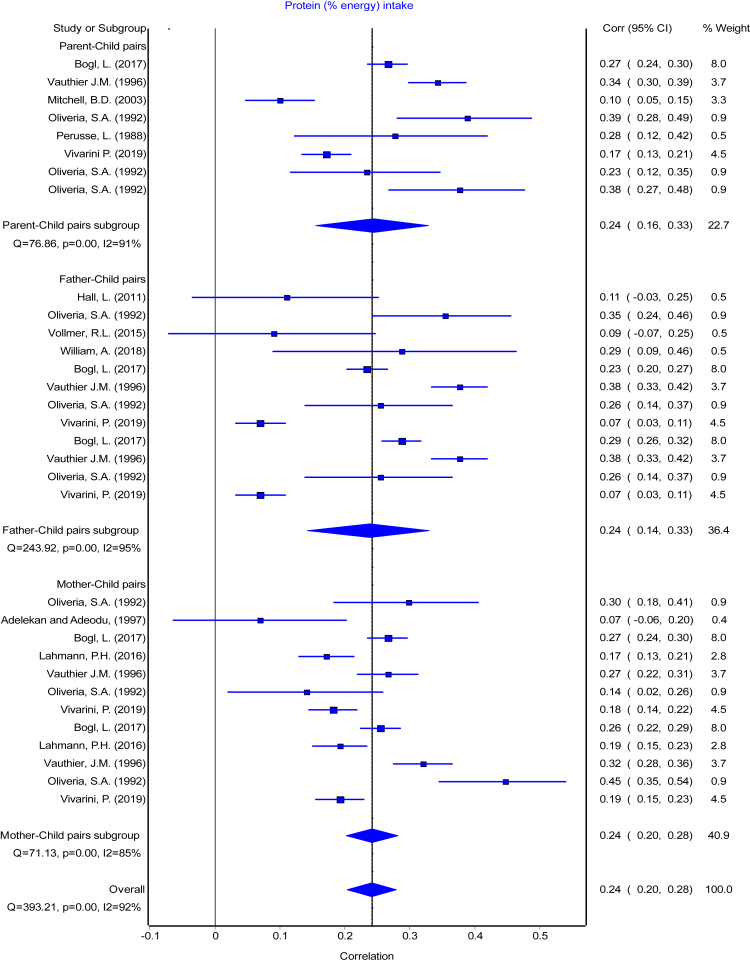
Forest plot of protein (% energy) intake. Comparing meta-analysis results of overall transformed correlation coefficient (z) on the association between parent and child resemblance on protein (% energy) intake in 45 studies for the three parents-child pairs. Pooled overall estimation with 32 data points for protein (% energy) intake–r: 0.24 (95% CI: 0.20, 0.28); test for heterogeneity: Q = 393.21, I^2^ = 92%, p < 0.01.

**Fig. 5:**
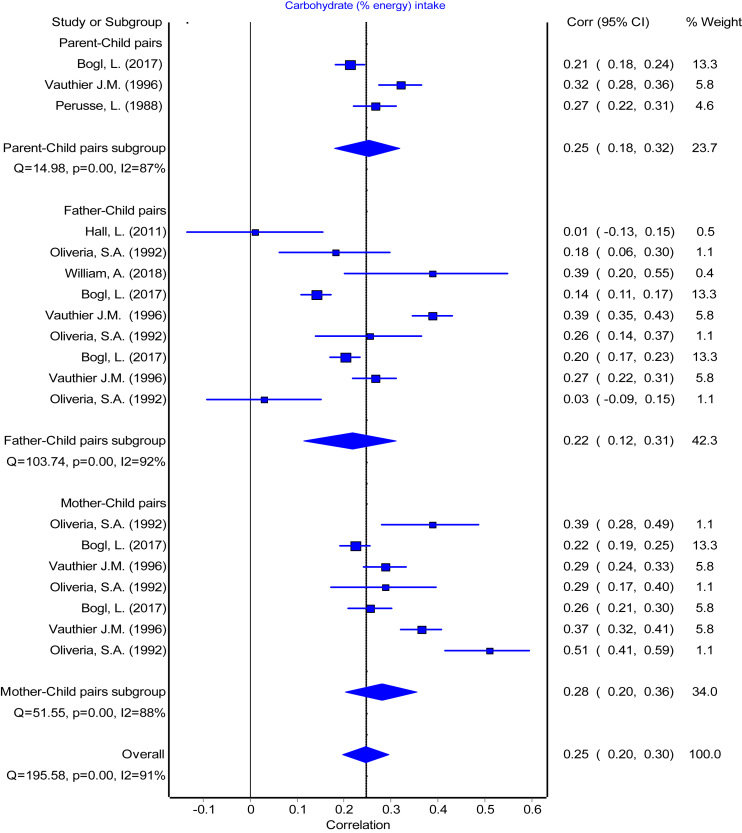
Forest plot of carbohydrate (% energy) intake. Comparing meta-analysis results of overall transformed correlation coefficient (z) on the association between parent and child resemblance on dietary intake in 45 studies for the three parents-child pairs. Pooled overall estimation from 19 data points for carbohydrate (% energy) intake r: 0.25 (95% CI: 0.20, 0.30); test for heterogeneity: Q = 196.58, I^2^ = 91%, p < 0.01.

**Fig. 6:**
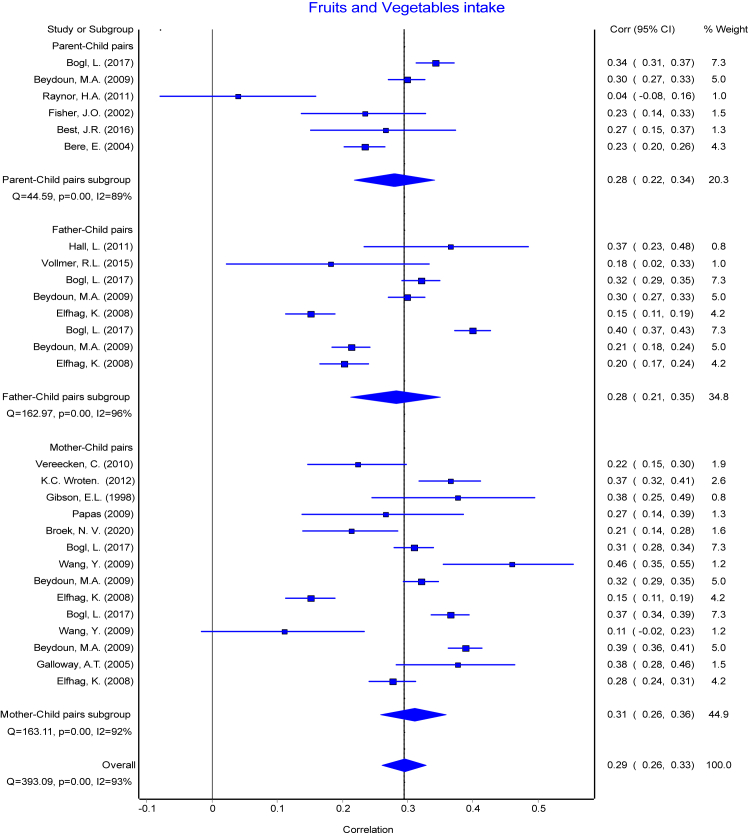
Forest plot of fruits and vegetables intake. Comparing meta-analysis results of overall transformed correlation coefficient (z) on the association between parent and child resemblance on dietary (fruits and vegetables) intake in 45 studies for the three parents-child pairs. Pooled overall estimation with 28 data points for fruits and vegetables intake–r: 0.29 (95% CI: 0.26, 0.33); test for heterogeneity: Q = 393.09, I^2^ = 93%, p < 0.01.

**Fig. 7:**
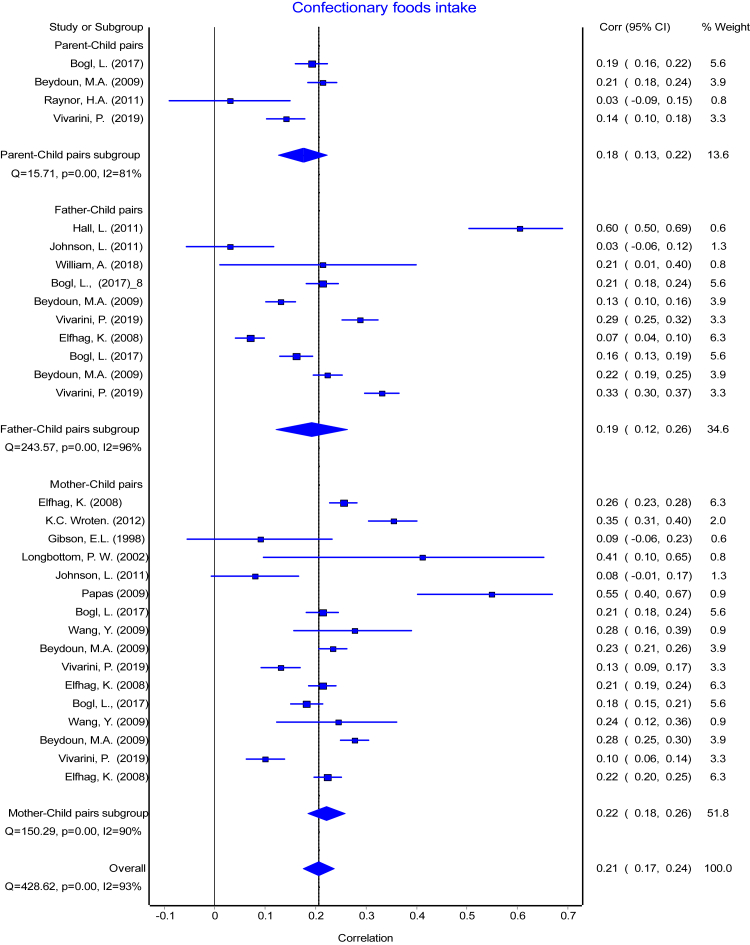
Forest plot of confectionary foods intake. Comparing meta-analysis results of overall transformed correlation coefficient (z) on the association between parent and child resemblance on dietary (confectionary) intake in 45 studies for the three parents-child pairs. Pooled overall estimation with 30 data points for confectionary foods intake–r: 0.21 (95% CI: 0.17, 0.24); test for heterogeneity: Q = 428.62, I^2^ = 93%, p < 0.01.

**Fig. 8:**
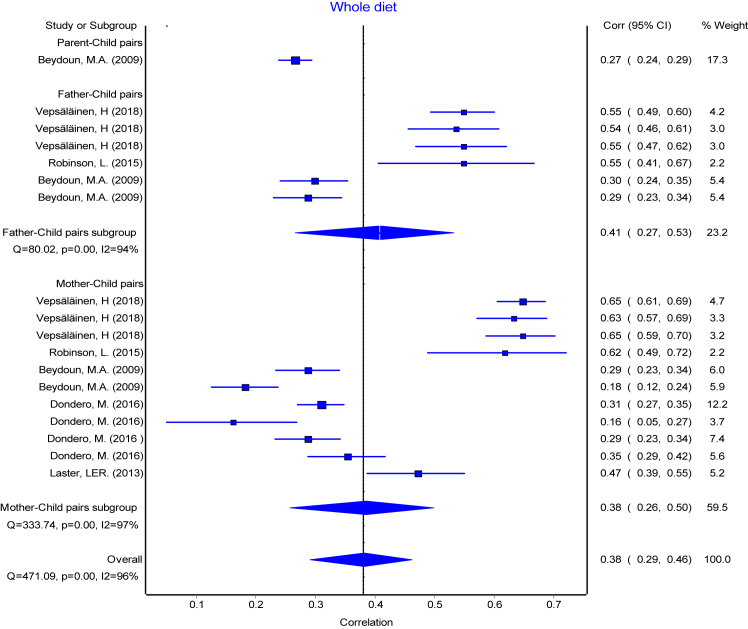
Forest plot of whole diet intake. Comparing meta-analysis results of overall transformed correlation coefficient (z) on the association of whole diet between parent and child resemblance in 45 studies for the three parents-child pairs. Pooled overall estimation with 18 data points for whole diet–r: 0.38 (95% CI: 0.29, 0.46); test for heterogeneity: Q = 471.09, I^2^ = 96%, p < 0.01.

The reported PC pairs resemblance ranged from −0.19 ^49^ to 0.57 ^17^ in the retrieved studies. The means of reported r with their 95% CI ranged from 0.19 to 0.32 and means of Fisher's transformed z ranged from 0.20 to 0.34 across the diet intake variables (Table 3). The association between each of the parent-child pairs (PC, FC, and MC) were pooled separately for each dietary intake and presented in forest plots (Fig. 2, Fig. 3, Fig. 4, Fig. 5, Fig. 6, Fig. 7, Fig. 8). Each figure shows considerable variation between studies but the pooled correlations for the three different PC pairs were similar with overlapping CIs within each dietary intake variables. The overall pooled z for PC pairs resemblance were weak across the dietary intakes. The pooled PC resemblance in ‘whole diet’ intake was higher than that for the traditional evaluation of individual intakes of nutrients and food items. All pooled r estimated from meta-analysis indicated significant heterogeneity and I^2^ ranged from 90% to 99%. Pooled transformed coefficients of seven dietary intakes varied widely across study characteristics (Table 3).

**Table 3:** Comparison of pooled transformed correlation coefficients of nutrients, food groups and whole diet by each study characteristics.

	Energy (kcal)	Fat (% kcal)	Protein (% kcal)	Carb (% kcal)	Fruits/Vegetables (g/d)	Confectionary foods (g/d)	Whole diet
**All (n = 214)**	**(n = 57)**	**(n = 30)**	**(n = 32)**	**(n = 19)**	**(n = 28)**	**(n = 30)**	**(n = 18)**
Mean (reported r)^a^ (95% CI)	0.19 (0.17: 0.22)	0.20 (0.14:0.26)	0.22 (0.19: 0.25)	0.24 (0.20: 0.27)	0.28 (0.25: 0.31)	0.20 (0.17: 0.22)	0.32 (0.27:0.38)
Fisher's transformed z^b^ (95% CI), p-value and Heterogeneity	0.20 (0.17: 0.22) Q = 737.34, p < 0.001, I^2^ = 92%	0.21 (0.15:0.27) Q = 1961.49, p < 0.001, I^2^ = 98.5%	0.23 (0.20: 0.26) Q = 350.08, p < 0.001, I^2^ = 91%	0.24 (0.20: 0.28) Q = 166.21, p < 0.001, I^2^ = 89.2%	0.29 (0.26: 0.32) Q = 336.91, p < 0.001, I^2^ = 92%	0.20 (0.17: 0.23) Q = 378.14, p < 0.001, I^2^ = 92%	0.34 (0.28:0.41) Q = 295.19, p < 0.001, I^2^ = 94%
**Population**^c^							
USA	0.16 (0.12)	0.07 (0.14)	0.27 (0.11)	0.28 (0.17)	0.27 (0.11)	0.25 (0.13)	0.29 (0.08)
European countries	0.22 (0.11)	0.30 (0.10)	0.30 (0.05)	0.27 (0.08)	0.31 (0.07)	0.18 (0.10)	0.59 (0.05)
Australia	0.18 (0.11)	0.21 (0.20)	0.16 (0.07)	0.19 (0.26)	0.23 (0.09)	0.25 (0.17)	0.58 (0.04)
Others	0.20 (0.15)	0.10 (0.15)	0.07 (0.00)	0.27 (0.00)	–	–	–
p-value	0.39	**<0.001∗∗**	**<0.001∗∗**	0.91	0.34	0.31	**<0.0001∗∗**
**Publication year**^c^							
1980s	0.16 (0.16)	–	–	0.26 (0.00)	–	–	–
1990s	0.21 (0.13)	0.34 (0.10)	0.30 (0.10)	0.29 (0.11)	0.37 (0.00)	0.09 (0.00)	–
2000s	0.17 (0.10)	0.07 (0.13)	0.10 (0.00)	–	0.29 (0.09)	0.25 (0.11)	0.26 (0.04)
2010s	0.17 (0.07)	0.22 (0.09)	0.19 (0.07)	0.16 (0.12)	0.26 (0.10)	0.20 (0.14)	0.48 (0.16)
p-value	0.57	**<0.0001∗∗**	**<0.05∗**	0.29	0.44	0.35	**<0.001∗∗**
**Dietary assessment**^c^							
FFQ	0.14 (0.11)	0.19 (0.12)	0.17 (0.07)	0.19 (0.26)	0.27 (0.12)	0.22 (0.17)	0.59 (0.05)
24 h recall	0.21 (0.05)	0.11 (0.12)	0.26 (0.19)	0.19 (0.03)	0.33 (0.05)	0.22 (0.06)	0.29 (0.08)
Food record	0.20 (0.10)	0.34 (0.14)	0.27 (0.11)	0.29 (0.11)	0.18 (0.08)	0.20 (0.14)	–
Mixed	0.11 (0.20)	–	0.14 (0.11)	–	0.29 (0.09)	0.09 (0.00)	–
Others	0.44 (0.00)	–	–	–	0.26 (0.00)	0.54 (0.00)	–
p-value	**<0.05∗**	**<0.001∗∗**	0.09	0.29	**<0.05∗**	0.11	**<0.0001∗∗**
**Diet data reported**^c^							
Self-completed	0.12 (0.18)	0.12 (0.11)	0.15 (0.07)	0.26 (0.00)	0.22 (0.07)	0.18 (0.09)	–
Mother	0.17 (0.11)	0.24 (0.16)	0.14 (0.06)	–	0.32 (0.08)	0.27 (0.25)	0.47 (0.00)
Mixed/Both	0.21 (0.09)	0.09 (0.00)	0.29 (0.08)	0.25 (0.12)	0.30 (0.11)	0.23 (0.12)	0.32 (0.14)
Others	0.14 (0.00)	–	0.10 (0.00)	–	0.36 (0.00)	0.35 (0.00)	0.59 (0.05)
p-value	0.13	0.17	**<0.001∗∗**	0.97	0.22	0.53	**<0.001∗∗**
**Study design**^c^							
Cross-sectional	0.17 (0.13)	0.18 (0.18)	0.17 (0.08)	0.22 (0.19)	0.27 (0.11)	0.21 (0.14)	0.42 (0.17)
Longitudinal	0.18 (0.10)	0.27 (0.10)	0.26 (0.10)	0.26 (0.11)	0.28 (0.08)	0.22 (0.12)	–
p-value	0.76	0.13	**<0.05∗**	0.54	0.74	0.75	–
**Child age group**^c^							
Childhood	0.19 (0.15)	0.02 (0.03)	0.15 (0.07)	0.13 (0.18)	0.25 (0.10)	0.22 (0.13)	0.57 (0.06)
Adolescence	0.18 (0.13)	0.08 (0.11)	0.13 (0.05)	–	0.23 (0.03)	0.54 (0.00)	–
Both	0.17 (0.10)	0.31 (0.10)	0.30 (0.07)	0.27 (0.11)	0.32 (0.10)	0.18 (0.07)	0.27 (0.06)
p-value	0.96	**<0.0001∗∗**	**<0.0001∗∗**	0.13	0.16	0.06	**<0.0001∗∗**
**Quality score**^c^							
Poor	0.21 (0.12)	0.05 (0.09)	0.14 (0.06)	0.01 (0.00)	0.31 (0.15)	0.25 (0.17)	0.46 (0.17)
Fair	0.16 (0.15)	0.19 (0.17)	0.15 (0.08)	0.27 (0.00)	0.23 (0.13)	0.21 (0.11)	0.28 (0.08)
Good	0.19 (0.09)	0.31 (0.11)	0.29 (0.08)	0.28 (0.11)	0.28 (0.10)	0.21 (0.10)	0.47 (0.00)
p-value	0.58	**<0.0001∗∗**	**<0.001∗∗**	0.10	0.29	0.79	0.14

∗ The pooled mean transformed correlation coefficient differed significantly within the study characteristics by one-way ANOVA (p ≤ 0.05).

∗∗ The pooled mean transformed correlation coefficient differed significantly within the study characteristics by one-way ANOVA (p < 0.001 and < 0.0001).

“–” denoted for no data point for the corresponding study characteristics.

a A total of 45 studies with 214 data points were used for meta-analysis. Values are pooled mean and 95% confidence interval of reported (r).

b Fisher's transformed correlation coefficient (z) for each dietary intake variables.

c Values are pooled mean and standard deviation of Fishers transformed correlation coefficient (z) by study characteristics for each nutrient, food groups, and whole diet.

In multivariable meta-regression models, we found energy intake resemblance among PC pairs was significantly associated with study population, publication year, dietary assessment methods, who reported the dietary data and child's age (Table 4). The association between PC pairs in energy intake in Australian studies (β ± SE: 0.89 ± 0.30, p < 0.01) in comparison with European studies (β ± SE: 0.34 ± 0.08, p < 0.001), and other countries (β ± SE: 0.31 ± 0.08, p < 0.001) showed significantly greater resemblance compared to studies in USA. The findings were similar for whole diet intake, however, differed for other dietary intake variables. We found studies published in the 1980s, 2000s, and 1990s, had significantly higher pooled mean PC pairs association in overall energy intake than studies published in recent decades, 2010s. However, publication year was not significantly associated with other dietary intake variables.

**Table 4:** Result of multivariable meta regression^a^: associated between parent-child resemblance of dietary intake and study characteristics based on Fisher's transformed correlations coefficient (z) from 45 studies^b^.

	Energy (kcal)		Fat (% kcal)		Protein (% kcal)		Carb (% kcal)		Fruits/Vegetables (g/d)		Confectionary (g/d)		Whole diet	
**Model 1**	β (SE)	p-value	β (SE)	p-value	β (SE)	p-value	β (SE)	p-value	β (SE)	p-value	β (SE)	p-value	β (SE)	p-value
**Population (ref** = **USA)**														
Europe	0.34 (0.08)	**<0.001**	−0.09 (0.05)	0.06	−1.86 (2.79)	0.51	−0.16 (0.15)	0.32	−0.15 (0.19)	0.46	−0.20 (0.15)	0.20	0.06 (0.07)	0.40
Australia	0.89 (0.30)	**<0.01**	−0.01 (0.14)	0.95	−1.16 (1.61)	0.48	0.41 (0.09)	**<0.01**	0.06 (0.10)	0.58	0.00 (0.09)	0.99	0.41 (0.03)	**<0.001**
Others	0.31 (0.08)	**<0.001**	−0.12 (0.14)	0.41	1.42 (1.87)	0.45	0.50 (0.13)	**<0.01**	–	–	–	–	–	–
**Publication year (ref ≥2010s)**														
1980s	0.74 (0.24)	**<0.01**	–	–	–	–	0	–	–	–	–	–	–	–
1990s	0.60 (0.17)	**<0.001**	−0.03 (0.08)	0.71	0.08 (0.29)	0.78	0.01 (0.05)	0.80	0.28 (0.24)	0.14	0.58 (0.29)	0.07	–	–
2000s	0.79 (0.28)	**<0.01**	0	–	−4.08 (5.38)	0.45	–	–	−0.14 (0.13)	0.28	−0.04 (0.05)	0.42	0.18 (0.07)	**<0.05**
**Dietary assessment methods (ref = food record)**														
FFQ	0.08 (0.07)	0.25	−0.11 (0.07)	0.15	1.09 (1.43)	0.45	0	–	0.33 (0.23)	0.18	0.44 (0.07)	**<0.001**	–	–
24 h recall	0.26 (0.08)	**<0.01**	−0.17 (0.07)	**<0.05**	0.97 (1.71)	0.57	0	–	0.38 (0.12)	**< 0.01**	0.11 (0.04)	**<0.01**	–	–
Mixed and Short questionnaire	0.29 (0.13)	**<0.05**	–	–	–	–	–	–	0.26 (0.16)	0.14	−0.06 (0.15)	0.67	–	–
**Diet data reported by (ref** = **mixed)**														
Self-completed	−0.23 (0.09)	**<0.01**	−0.06 (0.07)	0.37	0	–	0	–	0.04 (0.08)	0.57	−0.50 (0.05)	**<0.001**	–	–
Mother	0.13 (0.11)	0.25	–	–	−3.32 (4.97)	0.51	–	–	0.12 (0.18)	0.50	0.14 (0.08)	0.09	−0.16 (0.07)	**0.05**
Interviewer	−0.02 (0.08)	0.82	−0.28 (0.15)	0.07	–	–	–	–	0.05 (0.09)	0.59	0.36 (0.07)	**<0.001**		
**Study design (ref = longitudinal studies)**														
Cross-sectional	−0.01 (0.03)	0.70	0.22 (0.04)	**<0.001**	–	–	0	–	0.03 (0.06)	0.64	−0.83 (0.11)	**<0.001**	–	–
**Overall study quality**														
Quality score	0.57 (0.39)	0.15	1.28 (0.00)	**<0.001**	0.89 (0.18)	**<0.001**	1.66 (0.01)	**<0.001**	0.18 (0.34)	0.62	−1.70 (0.00)	–	0.00	–
**Model 2: Child age (mean)**														
**Age**	−0.02 (0.01)	**<0.001**	–	–	0.28 (0.41)	0.51	0.03 (0.01)	<0.01	0.01 (0.01)	0.58	0.00	–	−0.07 (0.01)	**<0.001**
**Model 3: Parent-child pairs (ignore: child sex; ref** = **parent-child)**														
Father-child	0.01 (0.03)	0.66	0.02 (0.03)	0.54	−0.01 (0.03)	0.69	−0.02 (0.03)	0.37	−0.01 (0.06)	0.98	0.03 (0.04)	0.46	−0.03 (0.06)	0.57
Mother-child	0.01 (0.02)	0.64	0.02 (0.01)	0.24	0.00 (0.02)	0.89	0.03 (0.04)	0.46	0.02 (0.05)	0.74	0.04 (0.05)	0.41	−0.01 (0.07)	0.88
Other pairs	–	–	–	–	0.01 (0.08)	0.88	–	–	–	–	–	–	–	–
**Model 4: Parent-child pairs (consider: child sex; ref** = **parent-child)**														
Parent-son	0.03 (0.06)	0.57	–	–	−0.05 (0.07)	0.49	0.00 (0.15)	0.97	−0.14 (0.08)	0.12	0	–	0.02 (0.06)	0.68
Parent-daughter	−0.04 (0.03)	0.22	−0.03 (0.02)	0.21	−0.02 (0.03)	0.38	−0.01 (0.05)	0.46	−0.03 (0.04)	0.39	−0.03 (0.04)	0.45	0.03 (0.05)	0.54
Other pairs	–	–	–	–	–	–	–	–	–	–	–	–	–	–

“–” denoted for values those were omitted because of collinearity within the variables.

∗Statistically significant at p < 0.05, p < 0.01, and p < 0.001 were highlighted as bold.

a Quality-effect weights were used for the meta-regression analysis.

b In all models, the outcome variables used in the meta-regression was the Fisher's transformed correlation coefficient (z). A Stata command “admetan” was used for the full models that included study population, publication year, dietary assessment method, diet data reported by, study design, study quality score as the independent variables. Model 2, 3 and 4 forced children's mean age and dummy variables of parent-child pairs as an additional predictor. In model 2, 3 and 4, Backward elimination was used and only predictors with p-values <0.10 were retained.

Dietary assessment method was a significant predictor of PC pairs resemblance. Compared to dietary data collected via food record, the 24-h recall method showed a higher pooled association for PC pairs resemblance in energy (kcal) (β ± SE: 0.26 ± 0.08, p < 0.01) and fruits/vegetables (β ± SE: 0.38 ± 0.12, p < 0.01) intake, respectively. However, fat (% energy) and confectionary foods (g/d) intake showed weaker PC pairs associations using the 24-h recall method. Data on who reported the diet was a significant predictor for energy (kcal/d), confectionary foods (g/d) and whole diet intake in model 1. Study design showed opposite effects in relation to fat and confectionary foods intake, while study quality scores were related to all three macronutrient intakes. Further, we tested whether the correlations differed by child's mean age, parent gender or child gender, these were entered as predictors in models 2, 3 and 4, respectively. We found a weaker association with parent intakes as child's mean age increased in energy (kcal/d) and whole diet intake in model 2. Apart from that, these characteristics showed no significant associations among different dietary intake variables.

No publication bias was observed in the Doi plots across studies for energy intake, fat (% energy), protein (% energy), carbohydrates (% energy), and confectionary foods intake in the Supplementary Figure S1 (appendix pp 29). However, minor asymmetry was present in the Doi plot for studies on fruits/vegetable intakes and the results of the LFK index also suggested minor negative asymmetry of the Doi plot (LFK index = −1.35). Additionally, risk of bias across studies for whole diet was visualized in a Doi plot, indicating an asymmetric shape for the pooled whole diet intake (Supplementary Figure S1h). The LFK index was 3.97, also indicating major publication bias for whole diet. The overall findings for publication bias might provide equivocal evidence across dietary intakes, implying that studies with diverse parent-offspring resemblance outcomes exist. However, major publication bias for whole diet might also be attributable to the few numbers of studies included in the meta-analyses.

## Discussion

In this systematic review and meta-analysis, consistent but weak to moderate positive associations were found for each dietary intake variables (energy, nutrients, food groups, and whole diet) for resemblance between PC pairs. Overall, these associations persisted in stratified and subgroup analyses. Our findings are similar to the previous evidence on dietary resemblance between parent-child pairs. A previous meta-analysis published by Wang et al. (2011), summarized the relationship between the parent and child dietary intakes in 24 studies^32^ concentrating only on energy and nutrient intakes (total fat and fat (% energy)), so missed many important studies particularly those reported food group intakes.^48^^,^^50^, ^51^, ^52^, ^53^, ^54^, ^55^, ^56^, ^57^ Additionally, the author also identified insufficient data points and limited statistical power due to relatively small sample sizes as limitations; therefore, recommended for the inclusion of additional food groups in future meta-analysis.^32^ Since then up to 31 December 2020, many additional articles (n = 37) have been published on PC pairs resemblance of dietary intakes and associated factors, thus warranting our updated evidence synthesis.

The evidence from the current review suggests that studies reporting resemblance using whole diet approaches showed relatively higher PC pairs associations compared to single nutrient intakes and food groups. Since whole diets are combinations of foods eaten by an individual and these foods deliver nutrients, they may give better reflections of real-life situations by giving attention to overall foods consumed.^17^ The whole diet approach between parents and children suggests that both may be exposed to combinations of similar food items, e.g., healthy foods^58^ and therefore show higher parent-child similarities.^59^ Our study notably adds to the existing body of evidence by summarizing the available articles on whole diet and finding a stronger association between parent and offspring when assessing the diet in this way. However, very few studies investigating PC pairs resemblance using whole diet were available and only four studies were included in the meta-analysis. Due to publication bias, no firm conclusion on the resemblance in whole diet intake can be drawn between parents and children. Therefore, we recommend that more research should investigate PC pairs resemblance using whole diet approaches.

In our review the transformed correlation coefficients between parent-child dietary intakes indicated considerable variability across different dietary intake variables, PC pairs and study characteristics. However, pooled correlations across dietary intake variables displayed in the forest plots suggested no substantial differences between FC and MC pairs, thus concluding that dietary resemblance between PC pairs was similar for both mother and father pairs. These findings are somewhat similar to a recent study^17^ and contrary to some other studies that have concluded that maternal dietary intakes are more influential for children than paternal diet.^15^ As a result, health professionals have tended to emphasize the importance of maternal influences on the development of children's dietary habits.^15^^,^^33^^,^^60^ It is possible that the presence of both parents may have a greater influence on the resemblance in dietary intakes than one parent alone.^61^ This may be a consequence of dual reinforcement from both parents and the result of shared meals within the family.^16^

We also found that the largest number of studies reporting PC resemblance of dietary intakes came from the USA, and that these studies largely report weaker associations than some other countries. This may be explained by country-specific food environment and meal patterns of parents and children in different geographical regions. The USA Federal government operates school food programs, thus, children regularly consume most of their daily meals including breakfast and lunch at school.^62^^,^^63^ The provision of school meals varies significantly throughout European countries^64^ and other countries, such as Australia. Australian children's meals during school hours are mainly supplied from home, purchased from a school tuckshop or from takeaways.^65^ Therefore, the higher PC dietary resemblance in Australia may be the result of a relatively high number of children taking lunches from home to school. Furthermore, only four studies considered different socio-economic backgrounds of families, limiting the opportunity to examine the variation in parent-child resemblance from a lower socioeconomic lens.

Our findings from meta-regression provided evidence of possible predictors that may explain the significant heterogeneity in the PC pairs dietary intake associations. Our review found that study population, publication year, different dietary assessment methods provided consistent levels for the associations between PC pairs for some of the dietary intake variables, though they diverged for others. For example, studies using 24-h recalls yielded higher positive associations in energy, and fruit and vegetable intakes, but inverse results for fat (% energy) than those using food records for the dietary assessment. In contrast, confectionary foods (g/d) intake showed significantly higher PC pairs associations with FFQ compared to 24-h recall. These results may be due to different biases between dietary assessment methods in levels of misreporting of dietary intakes.^66^ Evidence suggests that people who under report dietary intakes tend to report lower intakes of fat, confectionary foods (sugar-containing foods such as biscuits, cakes, chocolates) and sweets.^67^, ^68^, ^69^, ^70^

The finding also indicates the importance of considering who reported the food consumption information in the study as this may define the extent of association between PC intakes. We found a weaker association between PC pairs in energy intakes and confectionary food intakes if the dietary information was self-completed in comparison to an interviewer administered approach. This could be due to a tendency to report lower intakes in self-completed method.^71^, ^72^, ^73^ When using the mother as an informant, weaker associations in PC pairs were found for whole diet intake, but not for other dietary variables. We may speculate whether mothers tend to under report regarding the foods they prefer their children not to have eaten.^67^

This study observed mainly weak associations between PC pairs across dietary intake variables and there could be several possible interpretations. First, this might be the cumulative impact of modernized society where both parent and child spend long hours away from home. It is likely that children become more autonomous in their food intake when eating in absence of parents regularly.^74^ The modern lifestyle pattern with lack of quality time for families may affect eating behaviours of parents and children by having fewer shared family meals at home,^17^ eating outside the home more, and increasing reliance on takeaways or home delivery.^75^ Such transitions from traditional family meal patterns to modern eating habits may have important impacts on the diets of both children and parents^61^ and thus weaken parent-child dietary resemblance. Moreover, like other behavioural traits such as weight status,^76^, ^77^, ^78^ and physical activity level,^79^^,^^80^ it is likely that the shared home food environment has in the past led to dietary intake resemblance among parent-child pairs.

The key strengths of this review include the comprehensive nature of the inclusion criteria, assessment of risk bias, robust analysis, and the systematic examination of multiple dietary intakes including food groups and whole diet from 1980 to 2020. We adjusted for multiple confounders including who reported the dietary data, child's age and study design. This study should be interpreted in the light of some limitation. First, this review did not include latest published studies from 2021 to 2022 and there may be some relevant studies published since 2020. Therefore, we have carried out a scoping exercise to investigate this possibility by searching six electronic databases for published articles on parent-child resemblance in dietary intakes. We identified a total of 9386 published articles: from PubMed (5776), Embase (1645), CINAHL (681) Medline (57), APA PsycNet (27) and Web of Science (1200). In this preliminary search (before going for systematic screening), we identified only two papers from one study that merited further investigation (see in the supplementary appendix (Table S3.1, appendix pp 28)).

In the current systematic review, these two studies would only provide one data point to add to 4 dietary variables (energy, carbohydrates, protein, and whole diet). Also, both studies reported weak dietary resemblance using partial correlations, therefore, adding these data points would not change the conclusion of our review. There are further limitations to the current study, which reduce the generalisability of the findings. The first is the predominance of studies from the USA and European countries and in Caucasian populations. Second, we could not investigate the effects of socio-economic characteristics on the parent-child association of dietary consumption because studies used diverse socioeconomic status measures, and some did not assess this at all. Studies investigating PC pairs resemblance using overall dietary intake or whole diet approaches were limited in number. We are in the process of developing a manuscript which should add to the published data on PC pairs resemblance using whole diet.

Overall, findings from the present review question the societal belief that parental dietary behaviour has a central role in influencing dietary intakes among children. Individual food preferences, overall food environment, changes in lifestyle due to modernization and behavioural autonomy may have altered the degree of resemblance of dietary intakes within the family. More research is needed focusing on whole diet approaches to capture overall food consumption of parent-offspring. Moreover, parent-child associations using whole diet could be a useful approach that may shed some light on existing low to moderate evidence of resemblance in dietary intakes. Further rigorous research is needed focusing on PC pairs resemblance in diverse socio-demographic stratum, and family food environments. More studies in disadvantaged groups and people from low- and middle-income countries will help to elucidate potential differences in the PC resemblance in dietary intakes across different social contexts.

## Contributors

SP and AAM conceived, designed the study and were responsible to write the protocol. SP primarily extracted the data and TB verified extracted studies. Any difference in opinion were resolved by discussion among SP, TB, and AAM. Data analysis was performed by SP and verified by MMH and AAM. AAM and TB has direct access to the database. All authors (PE, NT, KN, DM, YF, TB, MMH and AAM) contributed to the interpretation of the results, participated in manuscript writing, critically reviewed the manuscript, and approved the final manuscript for submission.

## Data sharing statement

All extracted data supporting the findings of this specific systematic review and meta-analysis are available upon request after approval of a proposal from the corresponding author (SP; s.pervin@uq.edu.au).

## Declaration of interests

We declare no competing interests.
