# Supplementary material for: The myth and reality of familial resemblance in dietary intake: a systematic review and meta-analysis on the resemblance of dietary intake among parent and offspring
Source: eClinicalMedicine. 2023 Jun 2;60:102024. doi: 10.1016/j.eclinm.2023.102024 (PMC10251076; doi:10.1016/j.eclinm.2023.102024)
Supplement: Supplementary Figures S1–S9 and Tables S1–S4 [file mmc1.docx]

**Supplementary appendix**

**The myth and reality of familial resemblance in dietary intake: A systematic review and meta-analysis on the resemblance of dietary intake among parent and offspring**

Sonia Pervin ^a, b*^, Pauline Emmett ^c^, Nick Townsend ^d^, Tuhin Biswas ^b, e^, M Mamun Huda ^b, f^, Kate Northstone ^g^, Yaqoot Fatima ^b, f, h^, H. David McIntyre ^i^, Abdullah Al Mamun ^b, f^

^a^ Institute for Social Science Research, The University of Queensland, Brisbane, Australia

^b^ ARC Centre of Excellence for Children and Families over the Life Course, The University of Queensland, Brisbane, Australia

^c^ Centre for Academic Child Health, Population Health Sciences, Bristol Medical School, University of Bristol, Canynge Hall, 39 Whatley Road, Clifton, Bristol BS8 2PS

^d^ Centre for Exercise, Nutrition and Health Sciences, School for Policy Studies, University of Bristol, 8 Priory Rd, Bristol BS8 1TZ

^e^ Science and Math Program, Asian University for Women, Chattogram, Bangladesh.

^f^ Poche Centre for Indigenous Health, Faculty of Health and Behavioural Sciences, The University of Queensland, 74 High St, Toowong QLD 4066

^g^ Population Health Sciences, Bristol Medical School, University of Bristol, Oakfield House, Oakfield Grove, Bristol, BS8 2BN

^h^ Centre for Rural and Remote Health, James Cook University, Mount Isa, Queensland, Australia

^i^ Mater Clinical Unit and Mater Research, Faculty of Medicine, The University of Queensland, Raymond Terrace, South Brisbane, Queensland, 4101

* **Corresponding author:**

Sonia Pervin

Institute for Social Science Research

ARC Centre of Excellence for Children and Families over the Life Course

The University of Queensland

80 Meiers Road, Long Pocket Precinct, Indooroopilly, Queensland 4068, Australia

Tel + 61 470653442

Fax: + 61 7 334 67646

E-mail: [s.pervin@uq.edu.au](mailto:s.pervin@uq.edu.au)

**Supplementary texts: Data extraction**

The following keywords or medical subject headings on MEDLINE were used: (Resemblance OR association OR comparison OR tracking OR distribution OR similarity OR similar OR concordance OR relationship) AND (household? OR house-hold? OR familial OR parent* OR father* OR mother* OR paternal* OR maternal* OR generation OR child* OR offspring* OR adolescent*) AND (Food* OR nutrient* OR macronutrient* OR macro-nutrient* OR micronutrient* OR micro-nutrient* OR food OR diet* OR dietary* OR nutrition* nutritional* OR eating OR diet adj5 (factor* OR pattern* OR habit* OR consum* OR unhealthy OR healthy OR healthful) OR food preference OR food intake OR fast food OR fat OR carbonated beverage OR energy drink OR carbonated drink OR soft drink OR unhealthy food* OR healthy food* OR salt* OR sugar* OR carbohydrate* OR sodium* OR consum* OR vegetable? consum* OR whole diet* OR protein* OR energy* OR total fat* OR confectionary* food* OR fruit* OR vege* OR fruit* vege* OR snack* OR beverage* ) for relevant studies. The full details on the search strategy for all three databases are presented in the supplemental materials (Supplement Table 1). We restricted our search to the studies published in English and studies in human.

We included studies examining the association between parent and offspring on dietary resemblance through correlation coefficient (r), intraclass correlation coefficient and reported information on dietary intake (including nutrients, food groups and whole diet). Any observational studies, including cohort, case-control, and cross-sectional studies published in English, were eligible for inclusion in this review (Table 1). Articles published as case reports, qualitative reports, comments, letters, and reviews were not included. The population of interest was parents (either parent, at least one parent, both parents) and their offspring (child, adolescent, or pre-adult). We did not consider any age restriction for offspring or parents. We considered parental data that were obtained from either biological or non-biological parents.

The primary outcome of this review and meta-analysis is the resemblance of offspring’s dietary intake to their parent/s dietary intake. Parents' dietary intake was defined as the exposure variable, and the child’s intake defined as the outcome variable. Studies that reported associations between exposures and outcome were eligible for inclusion. A pilot review, completed before we carried out the main review, suggested several ways in which dietary exposure could be measured, such as using food frequency questionnaires (FFQ) or direct record of foods as consumed, along with a number of other tools. All were considered eligible and presented accordingly in this review. We included parental dietary intake considering both parents (father and mother) or any parent (father or mother) with their child’s dietary intake to assess the resemblance between parent and offspring through similarity or association or concordance.

SP and TB were responsible for assessing all titles and abstracts for inclusion using literature screening online software Rayyan independently ^1^, discussing all ambiguous records with each other. If TB did not reach 90% agreement with SP, a third reviewer (AAM) assessed the record. Furthermore, any remaining disagreements were discussed and resolved via study team (SP, TB, YF and AAM) consensus and SP reassessed all records until a 90% agreement was reached. A similar approach was used for full-text review. Study authors were contacted by e-mail if any included study had omitted relevant details. Studies with a lack of relevant information were ineligible for inclusion.

We developed a list of data collection checklist including multiple variables for extraction and received feedback from the study team on the draft. Two separate data extraction forms (summarized the overall study characteristics and extracted reported r by PC pair) were developed and piloted with five randomly selected studies to ensure standardization. We extracted data on the descriptive and quantitative characteristics of studies, exposure and outcome data, and different indicators of quality, including the following: publication details (e.g., title, objectives, year of publication, language, publication status), characteristics of study and population (e.g., study year, study design, methods, country, setting, sample size, source of the sample, age, gender, ethnicity, measures of socio-economic status, measures of family structure, parent-offspring dyad, who reported exposure and outcome, duration of the study, and study strength and limitation), details about the association (e.g., type of dietary intake variables, estimation by diet intake variables and by parent-child dyad, analysis and reported degree of association). Correlation coefficients such as Pearson or Spearman rank correlation coefficients and intra-class correlation coefficients were extracted as a measure of association.

The first author (SP) was responsible for extracting all information from included studies using the piloted form, discussing all inconsistencies with TB and a third reviewer where necessary. Simultaneously, a second person (TB) verified 20% of studies for general characteristics information and 100% of studies regarding exposure and outcome information. Any disagreements were resolved by consensus between SP and TB or by a third team member if needed. Any major discrepancies in data collection that SP, TB and a third reviewer failed to resolve were brought to a full group discussion and resolved accordingly. In addition, the third reviewer, AAM and DM, independently extracted data from a different 10% random samples to ensure a high degree of consistency.

**Supplementary table 1: Literature search strategy for data extraction from three online databases including, PubMed Search, Embase and CINAHL**

| **Database** | **Search strategies** |
| --- | --- |
| PubMed Search | ((Resemblance[Title] OR similarity[Title] OR resemble*[Title] OR similar[Title] OR concordance[Title] OR association[Title] OR tracking[Title] OR distribution[Title] OR comparison[Title] OR relationship[Title]) AND (household?[Title] OR 'house hold?'[Title] OR family[Title] OR families[Title] OR familial[Title] OR parent*[Title] OR father*[Title] OR mother*[Title] OR paternal*[Title] OR maternal[Title] OR generation[Title] OR child*[Title] OR offspring*[Title] OR adolescent*[Title] OR adoles*[Title])) AND (food*[Title] OR nutrient*[Title] OR nutrient*intake[Title] OR macronutrient*[Title] OR macro-nutrient*[Title] OR micronutrient*[Title] OR micro-nutrient*[Title] OR food[Title] OR diet*[Title] OR dietary*[Title] OR eating*[Title] OR eating*pattern*[Title] OR eating*habit*[Title] OR food*consum*[Title] OR food*pattern*[Title] OR dietary*factor*[Title] OR dietary*pattern*[Title] OR dietary*habit*[Title] OR dietary*consum*[Title] OR unhealthy*diet*[Title] OR healthy*diet*[Title] OR healthful*diet*[Title] OR food*preference*[Title] OR food*intake*[Title] OR fast*food*[Title] OR fat[Title] OR carbonated*beverage*[Title] OR energy*drink*[Title] OR carbonated*drink*[Title] OR soft*drink*[Title] OR unhealthy*food*[Title] OR healthy*food*[Title] OR salt*[Title] OR sugar*[Title] OR carbohydrate*[Title] OR sodium*[Title] OR consum*[Title] OR vegetable*?consum*[Title] OR whole*diet*[Title] OR protein*[Title] OR energy*[Title] OR totalfat*[Title] OR confectionary*food*[Title] OR fruit*[Title] OR vege*[Title] OR fruit*vege*[Title] OR snack*[Title] OR beverage*[Title]) AND ( "1980/01/01"[PDat] : "2020/12/31"[PDat] ) AND Humans[Mesh]) |
| Embase | resemblance:ti OR similarity:ti OR resemble*:ti OR similar:ti OR concordance:ti OR association:ti OR tracking:ti OR distribution:ti OR comparison:ti OR relationship:ti  AND  household?:ti OR 'house hold?':ti OR family:ti OR families:ti OR familial:ti OR parent*:ti OR father*:ti OR mother*:ti OR paternal*:ti OR maternal:ti OR generation:ti OR child*:ti OR offspring*:ti OR adolescent*:ti OR adoles*:ti  AND  food*:ti OR nutrient*:ti OR nutrientintake:ti OR macronutrient*:ti OR macro-nutrient*:ti OR micronutrient*:ti OR micro-nutrient*:ti OR food:ti OR diet*:ti OR dietary*:ti OR eating:ti OR eatingpattern:ti OR eatinghabit:ti OR foodconsum*:ti OR foodpattern:ti OR dietaryfactor*:ti OR dietarypattern*:ti OR dietaryhabit*:ti OR dietaryconsum*:ti OR unhealthydiet:ti OR healthydiet:ti OR healthfuldiet:ti OR foodpreference:ti OR foodintake:ti OR fastfood:ti OR fat:ti OR carbonatedbeverage:ti OR energydrink:ti OR carbonateddrink:ti OR softdrink:ti OR unhealthyfood*:ti OR healthyfood*:ti OR salt*:ti OR sugar*:ti OR carbohydrate*:ti OR sodium*:ti OR consum*:ti OR vegetable?consum*:ti OR wholediet*:ti OR protein*:ti OR energy*:ti OR totalfat*:ti OR confectionary*food*:ti OR fruit*:ti OR vege*:ti OR fruit*vege*:ti OR snack*:ti OR beverage*:ti  #7 AND #8 AND #9 AND [article]/lim AND ([male]/lim OR [female]/lim) AND [humans]/lim AND [english]/lim AND [embase]/lim AND [1980-2020]/py AND ([child]/lim OR [preschool]/lim OR [school]/lim OR [adolescent]/lim OR [adult]/lim) |
| CINAHL | TI (Resemblance OR similarity OR resemble* OR similar OR concordance OR association OR tracking OR distribution OR comparison OR relationship) AND TI ( household? OR 'house hold?' OR family OR families OR familial OR parent* OR father* OR mother* OR paternal* OR maternal OR generation OR child* OR offspring* OR adolescent* OR adoles* )  AND TI ( food* OR nutrient* OR nutrientintake OR macronutrient* OR macro-nutrient* OR micronutrient* OR micro-nutrient* OR food OR diet* OR dietary* OR eatin. OR eatingpattern OR eatinghabit OR foodconsum* OR foodpattern OR dietaryfactor* OR dietarypattern* OR dietaryhabit* OR dietaryconsum* OR unhealthydiet OR healthydiet OR healthfuldiet OR foodpreference OR foodintake OR fastfood OR fat OR carbonatedbeverage OR energydrink OR carbonateddrink OR softdrink OR unhealthyfood* OR healthyfood* OR salt* OR sugar* OR carbohydrate* OR sodium* OR consum* OR vegetable?consum* OR wholediet* OR protein* OR energy* OR totalfat* OR confectionary*food* OR fruit* OR vege* OR fruit*vege* OR snack* OR beverage*)  Limiters - Published Date: 19800101-20201231; English Language; Human; Publication Type: Journal Article |

**Supplementary table 2: Quality assessment of studies included in the systematic review and meta-analysis**

| **Study** | **Design Bias^1^** | | **Selection Bias^2^** | | | | **Information Bias^3^** | | | | | **Confounding^4^** | **Analysis Bias^5^** | | **Total**  **Score** | **Max^6^**  **Score** | **Max**  **Quality**  **Score** |
| --- | --- | --- | --- | --- | --- | --- | --- | --- | --- | --- | --- | --- | --- | --- | --- | --- | --- |
|  | **Study Design** | **Follow up** | **Inclusion/**  **Exclusion criteria** | **Recruitment strategy** | **Interval between exposure & outcome assessment** | **Attrition** | **Pre-specified** | **Outcome assessment** | **Reliability of the outcome** | **Data collection and assessment** | **Exposure assessment** | **Confounding adjustment** | **Effect size** | **Data Availability** |  |  |  |
|  |  |  |  |  |  |  | **Outcome** |  |  |  |  |  |  |  |  |  |  |
|  | 1 | 2 | 3 | 4 | 5 | 6 | 7 | 8 | 9 | 10 | 11 | 12 | 13 | 14 |  |  |  |
| Vepsäläinen et al. 2018 | 0 | 0 | 1 | 1 | 0 | 0 | 1 | 0 | 0 | 1 | 1 | 3 | 1 | 1 | 10 | 17 | 0·59 |
| Bogl et al. 2017 | 2 | 1 | 1 | 1 | 1 | 1 | 1 | 1 | 1 | 1 | 1 | 3 | 1 | 1 | 17 | 17 | 1·00 |
| Lahmann et al. 2016 | 0 | 0 | 1 | 1 | 0 | 0 | 1 | 0 | 0 | 1 | 1 | 3 | 1 | 1 | 10 | 17 | 0·59 |
| Cullen et al. 2002 | 0 | 0 | 0 | 0 | 0 | 1 | 0 | 0 | 1 | 0 | 1 | 3 | 1 | 1 | 8 | 17 | 0·47 |
| Robinson et al. 2015 | 0 | 0 | 1 | 1 | 0 | 0 | 1 | 0 | 0 | 1 | 1 | 3 | 1 | 1 | 10 | 17 | 0·59 |
| Wang et al. 2009 | 2 | 1 | 1 | 1 | 0 | 1 | 1 | 0 | 0 | 0 | 1 | 3 | 1 | 1 | 13 | 17 | 0·76 |
| Beydoun et al. 2009 | 0 | 0 | 1 | 0 | 1 | 0 | 1 | 0 | 0 | 1 | 1 | 3 | 1 | 1 | 10 | 17 | 0·59 |
| Ovaskainen et al. 2009 | 2 | 1 | 1 | 1 | 1 | 1 | 1 | 1 | 1 | 1 | 1 | 3 | 1 | 1 | 17 | 17 | 1·00 |
| Feunekes et al. 1997 | 2 | 1 | 1 | 1 | 1 | 1 | 1 | 0 | 0 | 1 | 1 | 3 | 1 | 1 | 15 | 17 | 0·88 |
| Vereecken et al. 2010 | 2 | 1 | 1 | 1 | 1 | 1 | 1 | 1 | 0 | 0 | 1 | 3 | 1 | 1 | 15 | 17 | 0·88 |
| Billon et al. 2002 | 2 | 1 | 1 | 1 | 1 | 1 | 1 | 0 | 0 | 1 | 1 | 3 | 1 | 1 | 15 | 17 | 0·88 |
| Stafleu et al. 1994 | 0 | 0 | 1 | 1 | 1 | 1 | 1 | 1 | 1 | 1 | 1 | 3 | 1 | 1 | 14 | 17 | 0·82 |
| Wroten et al. 2012 | 0 | 0 | 1 | 1 | 1 | 0 | 1 | 1 | 1 | 1 | 1 | 3 | 1 | 1 | 13 | 17 | 0·76 |
| Vauthier et al. 1996 | 2 | 1 | 1 | 1 | 1 | 1 | 1 | 1 | 1 | 1 | 1 | 3 | 1 | 1 | 17 | 17 | 1·00 |
| Mitchell et al. 2003 | 2 | 1 | 1 | 1 | 1 | 1 | 1 | 1 | 1 | 1 | 1 | 3 | 1 | 1 | 17 | 17 | 1·00 |
| Hebestreit et al. 2017 | 2 | 1 | 1 | 1 | 1 | 1 | 1 | 1 | 0 | 1 | 1 | 1 | 1 | 1 | 14 | 17 | 0·82 |
| Grimm et al. 2004 | 0 | 0 | 1 | 1 | 0 | 0 | 1 | 0 | 0 | 1 | 1 | 1 | 1 | 1 | 8 | 17 | 0·47 |
| Gibson et al. 1998 | 0 | 0 | 1 | 1 | 0 | 0 | 1 | 0 | 0 | 0 | 1 | 3 | 1 | 1 | 9 | 17 | 0·53 |
| Hall et al. 2011 | 0 | 0 | 1 | 0 | 0 | 0 | 1 | 1 | 1 | 1 | 1 | 2 | 1 | 1 | 10 | 17 | 0·59 |
| Dondero et al. 2016 | 0 | 0 | 1 | 1 | 1 | 0 | 1 | 1 | 1 | 1 | 1 | 3 | 1 | 1 | 13 | 17 | 0·76 |
| Fisher et al. 2001 | 0 | 0 | 1 | 1 | 0 | 0 | 1 | 1 | 1 | 0 | 1 | 3 | 1 | 1 | 11 | 17 | 0·65 |
| Reinaerts et al. 2007 | 1 | 0 | 0 | 0 | 0 | 1 | 1 | 1 | 0 | 1 | 0 | 3 | 1 | 1 | 10 | 17 | 0·59 |
| Oliveria et al. 1992 | 2 | 1 | 1 | 1 | 1 | 1 | 1 | 1 | 1 | 1 | 1 | 3 | 1 | 1 | 17 | 17 | 1.00 |
| Raynor et al. 2011 | 0 | 0 | 1 | 1 | 0 | 0 | 1 | 1 | 0 | 1 | 1 | 3 | 1 | 1 | 11 | 17 | 0.65 |
| Cameron et al. 2011 | 1 | 0 | 0 | 1 | 1 | 0 | 1 | 1 | 0 | 1 | 1 | 0 | 1 | 1 | 9 | 17 | 0.53 |
| Fisher et al. 2002 | 2 | 1 | 1 | 1 | 0 | 1 | 1 | 0 | 1 | 0 | 1 | 3 | 1 | 1 | 14 | 17 | 0.82 |
| Longbottom et al. 2002 | 2 | 1 | 1 | 1 | 1 | 1 | 1 | 0 | 0 | 1 | 1 | 3 | 1 | 1 | 15 | 17 | 0.88 |
| Best et al. 2016 | 2 | 0 | 1 | 1 | 1 | 1 | 1 | 0 | 0 | 1 | 1 | 3 | 1 | 1 | 14 | 17 | 0.82 |
| Perusse et al. 1988 | 0 | 0 | 1 | 1 | 1 | 1 | 1 | 0 | 1 | 1 | 1 | 3 | 1 | 1 | 13 | 17 | 0.76 |
| Vollmer et al. 2015 | 0 | 0 | 1 | 1 | 0 | 0 | 1 | 1 | 1 | 1 | 1 | 3 | 1 | 1 | 12 | 17 | 0.71 |
| Galloway et al. 2005 | 1 | 1 | 1 | 1 | 1 | 1 | 1 | 0 | 1 | 1 | 1 | 3 | 1 | 1 | 15 | 17 | 0.88 |
| Laskarzewski et al. 1980 | 0 | 0 | 1 | 1 | 0 | 0 | 1 | 1 | 1 | 1 | 1 | 3 | 1 | 1 | 12 | 17 | 0.71 |
| Johnson et al. 2011 | 1 | 1 | 1 | 1 | 1 | 1 | 1 | 1 | 0 | 1 | 1 | 3 | 1 | 1 | 15 | 17 | 0.88 |
| Hannon et al. 2003 | 2 | 0 | 1 | 1 | 0 | 1 | 1 | 1 | 0 | 1 | 1 | 1 | 1 | 1 | 12 | 17 | 0.71 |
| Murrin et al. 2015 | 1 | 1 | 1 | 0 | 1 | 1 | 1 | 0 | 0 | 0 | 1 | 3 | 1 | 1 | 12 | 17 | 0.71 |
| Lipsky et al. 2019 | 0 | 1 | 1 | 1 | 0 | 1 | 1 | 0 | 1 | 1 | 1 | 3 | 1 | 1 | 13 | 17 | 0.76 |
| Stanton et al. 2003 | 2 | 1 | 1 | 1 | 0 | 1 | 1 | 0 | 0 | 0 | 1 | 3 | 1 | 1 | 13 | 17 | 0.76 |
| Patterson et al. 1988 | 0 | 0 | 1 | 1 | 0 | 1 | 1 | 0 | 1 | 1 | 1 | 3 | 1 | 1 | 12 | 17 | 0.71 |
| Vivarini et al. 2019 | 0 | 1 | 1 | 1 | 1 | 1 | 1 | 1 | 1 | 1 | 1 | 1 | 1 | 1 | 13 | 17 | 0.76 |
| Elfhag et al. 2008 | 2 | 1 | 1 | 1 | 1 | 1 | 1 | 0 | 0 | 1 | 1 | 3 | 1 | 1 | 15 | 17 | 0.88 |
| Bere et al. 2004 | 0 | 0 | 1 | 1 | 1 | 1 | 1 | 0 | 0 | 1 | 1 | 1 | 1 | 1 | 10 | 17 | 0.59 |
| Rossow et al. 1994 | 0 | 0 | 1 | 1 | 0 | 0 | 1 | 0 | 0 | 1 | 1 | 3 | 1 | 1 | 10 | 17 | 0.59 |
| Adelekan et al. 1997 | 0 | 0 | 1 | 1 | 0 | 0 | 1 | 0 | 0 | 1 | 1 | 3 | 0 | 1 | 9 | 17 | 0.53 |
| Feunekes et al. 1998 | 0 | 0 | 1 | 0 | 1 | 0 | 1 | 0 | 1 | 1 | 1 | 0 | 1 | 1 | 8 | 17 | 0.47 |
| Runyan et al. 2003 | 1 | 0 | 1 | 0 | 1 | 0 | 1 | 1 | 0 | 1 | 1 | 2 | 1 | 1 | 11 | 17 | 0.65 |
| Park et al. 2004 | 0 | 0 | 1 | 0 | 0 | 0 | 0 | 0 | 1 | 0 | 1 | 3 | 1 | 1 | 8 | 17 | 0.47 |
| da Veiga et al. 2006 | 0 | 0 | 0 | 0 | 0 | 0 | 1 | 0 | 0 | 0 | 1 | 2 | 1 | 1 | 6 | 17 | 0.35 |
| López et al. 2007 | 1 | 0 | 0 | 1 | 1 | 0 | 1 | 0 | 0 | 1 | 0 | 2 | 1 | 1 | 9 | 17 | 0.53 |
| Papas et al. 2009 | 2 | 1 | 1 | 1 | 1 | 1 | 1 | 0 | 0 | 1 | 1 | 3 | 1 | 1 | 15 | 17 | 0.88 |
| Rozin et al. 1991 | 1 | 0 | 0 | 0 | 1 | 0 | 1 | 0 | 0 | 1 | 0 | 2 | 1 | 1 | 8 | 17 | 0.47 |
| Hart et al. 2010 | 0 | 0 | 1 | 1 | 0 | 0 | 1 | 1 | 1 | 1 | 1 | 3 | 1 | 1 | 12 | 17 | 0.71 |
| Kunaratnam, K. et al, 2018 | 2 | 1 | 1 | 1 | 1 | 1 | 1 | 1 | 1 | 1 | 1 | 3 | 1 | 1 | 17 | 17 | 1.00 |
| Prichard, I, 2012 | 1 | 0 | 1 | 0 | 0 | 0 | 1 | 0 | 0 | 1 | 1 | 3 | 1 | 1 | 10 | 17 | 0.59 |
| Tada, Y, 2015 | 1 | 0 | 1 | 0 | 0 | 0 | 1 | 0 | 0 | 1 | 1 | 3 | 1 | 1 | 10 | 17 | 0.59 |
| Broek, N.Van, 2020 | 1 | 1 | 1 | 1 | 0 | 0 | 1 | 0 | 0 | 1 | 1 | 3 | 1 | 1 | 12 | 17 | 0.71 |
| Thorsdottir, I, 2006 | 2 | 1 | 1 | 1 | 1 | 0 | 1 | 1 | 1 | 1 | 1 | 2 | 1 | 1 | 15 | 17 | 0.88 |
| Laster, LER, 2013 | 2 | 1 | 1 | 1 | 1 | 1 | 1 | 1 | 1 | 1 | 1 | 3 | 1 | 1 | 17 | 17 | 1.00 |
| Vanhala, ML, 2010 | 1 | 1 | 1 | 0 | 1 | 1 | 1 | 1 | 1 | 1 | 1 | 2 | 1 | 1 | 14 | 17 | 0.82 |
| Harris, TS. Et al, 2015 | 1 | 0 | 1 | 1 | 1 | 0 | 1 | 1 | 1 | 1 | 1 | 3 | 1 | 1 | 14 | 17 | 0.82 |
| Williams, A., 2018 | 2 | 1 | 1 | 1 | 1 | 1 | 1 | 1 | 1 | 1 | 1 | 0 | 1 | 1 | 14 | 17 | 0.82 |
| Zuercher, JL., 2011 | 1 | 0 | 0 | 0 | 0 | 0 | 1 | 1 | 1 | 1 | 1 | 1 | 0 | 1 | 8 | 17 | 0.47 |

The details of the quality Assessment tool for observational cohort and cross-sectional studies developed by the National Institute of Health (NIH) are shown below:

**Total Scoring Criteria:**

**^1^Design bias**

1. What was the type of design? For prospective cohort scored 2 points, for retrospective cohort or case control scored 1 point, for cross-sectional studies scored 0 points.

2. Was the duration of follow-up appropriate for the demonstration of study outcome i.e., obesity (e.g., >= 12 months), scored 1 point for yes, scored 0 points for no or not applicable.

**^2^Selection Bias**

3. Did the inclusion/exclusion criteria remain consistent across the comparison groups of the study? Scored 1 points for yes, scored 0 points for no or not applicable.

4. Was the strategy for recruitment into the study the same across comparison groups (e.g., from same populations or both groups were recruited over the same time period)? Scored 1 points for yes, scored 0 points for no or when no information was provided.

5. Was the interval between the start of sleep exposure and the outcome was same across comparison groups, or if different, were appropriate analyses used to equalize this (e.g., time-to-event analyses)? Scored 1 points for yes, scored 0 points for no or not applicable or when no information was provided.

6. Was attrition < 20%, or if not, was follow-up done for these subjects to ensure their loss was not related to outcome? Scored 1 points for yes, scored 0 points for no or not applicable or when no information was provided.

**^3^Information Bias**

7. Were the outcomes of interest in the study pre-specified? Scored 1 points for yes, scored 0 points for no or when no information was provided.

8. Were reproducible measures (i.e., not self-reported) of study obesity outcomes implemented in the same way across comparison groups? Scored 1 points for yes, scored 0 points for no or when no information was provided.

9. Were there any safeguards described and used for assuring the reliability of study outcomes (e.g., recorded by a trained person, duplicate measurements, independent verification etc.)? Scored 1 points for yes, scored 0 points for no or when no information was provided.

10. Were data assessed and recorded in the same way for both comparison groups and across time points. Scored 1 points for yes, scored 0 points for no or when no information was provided.

11. Were exposures (or non-exposure) clearly defined (all essential components were described) and were assessed using objective tools or validated questionnaires in the same way across both study groups? Scored 1 points for yes, scored 0 points for no or when no information was provided.

**^4^Confounding Bias**

12. Were the groups similar at baseline in key confounding variables or if not, were steps taken to achieve comparability of key confounders (e.g., through matching, stratification, interaction terms, multivariate analysis, or other statistical adjustment such as instrumental variables)? If four or more confounders accounted for scored three points, if 2-3 confounders accounted for scored two points, if at least one major confounder accounted for scored 1 point, for unadjusted analysis scored 0 points

**^5^Analysis Bias**

13. Were effect sizes based on the data available at post assessment or pre-defined subgroups rather than a post hoc portion of the data? Scored 1 points for yes, scored 0 points for no or when no information was provided.

14. Were all data available (i.e., did not need to be estimated from results)? Scored 1 points for yes, scored 0 points for no or when no information was provided.

^6^Maximum score=17

**Supplementary table 3: Summary of the main characteristics and findings of the 61 studies that reported parent-child resemblance in dietary intake since 1980 to 2020**

| **Ref no** | **Authors (year), Country** | **Study type** | **Sample size** | **Child age; mean (median/ SD/range)** | **Parent’s details** | **Dietary**  **assessment** | **Dietary variables** | **Who Reported dietary intake** | **Main Findings** | **Reported resemblance (Overall)** |
| --- | --- | --- | --- | --- | --- | --- | --- | --- | --- | --- |
| 1 | ^2^Vepsäläinen et al. (2018)  Finland | Cross-sectional | 798 | 3-6 years | 29-44 years | FFQ, 47 items | Whole diet | Parent and legal guardians | Mother-child resemblance (Avg): 0.57; Father-child resemblance: 0.50 | Moderate |
| 2 | ^3^Bogl, et al. (2017)  8 European countries: Belgium, Cyprus, Estonia, Germany, Hungary, Italy, Spain and Sweden | Cohort | 3560 | 2–19 years | 25-65 years | 24-h recall | 4 macro-nutrients and 13 food groups | Parent reported for children below 11 years; Children reported by themselves aged 10 or younger with parental assistance; sibling assisted too | Parent–offspring (r = 0.11–0.33); Parent–offspring correlations were stronger for the intake of healthy (r = 0.33) than unhealthy (r = 0.10) foods | Strong |
| 3 | ^4^Lahmann, et al. (2016)  Australia | Cross-sectional | 2017 | 18 years | 14-46 years | FFQ | Food groups | Mother | Mother-offspring correlations were weak (r = 0.12–0.29); Partial correlation coefficient: Mother-daughter dyads (n = 1122)  **Total energy and Nutrients:** Energy, kcal: 0.15; Protein, g:0.19; Carbohydrate, (g): 0.21; Fat, (g): 0.24; Cholesterol, g: 0.25; Fiber, (g): 0.17; Sugar, g: 0.16; Alcohol, g: 0.14; Folate, mg: 0.18; Vitamin C, mg: 0.18; β-Carotene, mg: 0.21; Vitamin E, mg: 0.16; Retinol, mg: 0.21; Calcium, mg: 0.18; Iron, mg: 0.11; Magnesium, mg: 0.21 Sodium, mg; Zinc, mg: 0.17.  **Food groups, g:** Vegetables: 0.26; Legumes: 0.15; Potato: 0.17; Fruit: 0.16; Fruit juice:0.18; Dairy products: 0.12; Milk: 0.13; Eggs :0.21; Poultry :0.15; Red meat :0.14; Processed meat:0.11; Breakfast cereals :0.11; Cereal products: 0.07; Rice:0.28; Pasta or noodles : 0.13; Bread: 0.17; Spreads containing fat :0.17; Non-fat spreads:0.11; Nuts: 0.21; Snacks: 0.16; Cakes and pastries :0.07; Chocolate and sweets: 0.10. | Weak |
| 4 | ^5^Cullen, et al. (2002)  USA | Cross-sectional | 132 | 10 years | 33-47 years | Food record | Fat and Fruit, Juice, and Vegetable Practices (FJVP) | Children and Parent | Child LFPs were significantly correlated with parental low-fat practices (r=0.28); fat substitution (r=0.24); and fruit, juice, and vegetable practice (r=0.21) | Weak |
| 5 | ^6^Robinson, et al. (2015)  Australia | Cross-sectional | 122 | child aged 8–12 years | 35-50 years | FFQ, 120-item | RED foods; Fruits and vegetables | Children (Parent assisted) and Parent | Mother–child dyads, r = 0.27–0.47. Father–child dyads, r = 0.01–0.52.  Energy (kJ day): 0.47; Protein (% energy): 0.61; Carbohydrate (% energy):0.51; Total fats (% energy): 0.22; Saturated fats (% energy): 0.21; Core foods (% energy): 0.45; Noncore foods (% energy): 0.45; Alcohol (% energy): 0.65; Fibre (g 1000 kJ 1): 0.30; Diet quality (ARFS/ACARFS): 0.59; Vegetables: 0.73; Fruit: 0.65; Meat: 0.39; Vegetarian protein sources: 0.21; Grains: 0.42; Dairy: 0.40; Condiments: 0.13; Water: 0.20 | Weak-to-moderate |
| 6 | ^7^Wang, et al. (2009)  USA | Cross-sectional | 242 | 10-14 years | 30-50 years | Mixed: FFQ Youth and Adolescent Questionnaire (YAQ); FFQ | Nutrient intake (fat, fiber and calcium) and food groups (fruits and vegetables, fried food, sweetened beverages, snacks) | Child and Parent | Energy (kcal): Mother-daughter: 0.26; Mother-son: -0.24; Mother-child: 0.04 Fat (g): Mother-daughter: 0.30; Mother-son: -0.21; Mother-child: 0.07. Fat (% energy): Mother-daughter: 0.11; Mother-son: 0.19; Mother-child: 0.16. Fiber (g): Mother-daughter: 0.12; Mother-son: 0.08; Mother-child: 0.02. Calcium (mg): Mother-daughter: 0.19; Mother-son: -0.19; Mother-child: 0.02 | Weak |
| 7 | ^8^Beydoun, et al. (2009)  USA | Cross-sectional | 4244 | 2-18 years | 20–65 years | 2 X 24h recall | Healthy Eating Index score (HEI). | Mixed: Child and Parent | Parent–child, r: 0.20–0.33; Total dietary quality score: Parent-child: 0.26; Mother-daughter: 0.18; Mother-son: 0.28; Father-daughter: 0.28; Father-son: 0.29. **Energy (kcal)**: Parent-child: 0.22; Mother-daughter: 0.26; Mother-son: 0.23; Father-daughter: 0.14; Father-son: 0.29.  **Fat (g)**: Parent-child: 0.24; Mother-daughter: 0.24; Mother-son: 0.28; Father-daughter: 0.18; Father-son: 0.27.  **Fat (% energy)**: Parent-child: 0.01; Mother-daughter: 0.02; Mother-son: 0.04; Father-daughter: 0.02; Father-son: 0.01. Vegetable and Fruits: Parent-child: 0.29; Mother-daughter: 0.37; Mother-son: 0.31; Father-daughter: 0.21; Father-son: 0.29; Spearman correlation: Fruit: 0.26; Vegetables: 0.40; Snacks/desserts: 0.50; Meats: 0.29; Dairy: 0.18; Soda: 0.25. | Weak-to-moderate |
| 8 | ^9^Ovaskainen, et al, (2009)  Finland | cross-sectional | 2134 | 1-6 years | 18-39 years | Mixed: Child- 3-day food records; Mother: FFQ, 181-item FFQ | Dietary pattern clustering | Mixed: Parent; day carer | Familial dependence on dietary clusters was observed in mother–child pairs of 6-year-old children (P=0·035); but not in younger children (1 year). In 6-year-old children, the cluster ‘healthy, low-fat’ had a high proportion in the clusters ‘fat conscious’ and ‘modern, healthy’ of mothers. A higher frequency was observed for children being members of the cluster ‘fast food, sweet’ when mother belonged to the cluster ‘fast food, plenty’, ‘refined, sugar and butter’ or ‘sweet, fast food’. At 3 years of age, marginal significance of familiarity (P=0·054) among child–mother dyads. At the age of 1 year, no indication of familiarity was observed. | Weak-to-moderate |
| 9 | ^10^Feunekes, et al., (1997)  Netherlands | Repeated cross-sectional | 4030 | 1-3 years | 30-50 years | 2-day dietary record | Nutrient intake (Energy, Total fat, SF, MUFA, PUFA, cholesterol) | Mixed: Self-reported, mostly; for children aged <=13 y by parents | Father-son: Energy (MJ): 0.19; Total fat (% energy): 0.40; Saturated fat (% energy): 0.43; MUFA (% energy): 0.38; PUFA (% energy): 0.50; Cholesterol (mg/MJ): 0.41; Father-daughter: Energy (MJ): 0.24; Total fat (% energy): 0.39; Saturated fat (% energy): 0.37; MUFA (% energy): 0.42; PUFA (% energy): 0.48; Cholesterol (mg/MJ): 0.46. Mother-son: Energy (MJ): 0.09; Total fat (% energy): 0.37; Saturated fat (% energy): 0.43; MUFA (% energy): 0.38; PUFA (% energy): 0.50; Cholesterol (mg/MJ): 0.47; Mother-daughter: Energy (MJ): 0.24; Total fat (% energy): 0.44; Saturated fat (% energy): 0.45; MUFA (% energy): 0.45; PUFA (% energy): 0.50; Cholesterol (mg/MJ): 0.55. | Moderate to strong |
| 10 | ^11^Vereecken, et al., (2010)  Belgium, East and West Flanders | Longitudinal study | 609 | 10 years | 40-59 years | FFQ | Fruit, vegetables, sugared soft drinks, sweets, and crisps (FFQ), F&V score; excess score | Self-reported under the supervision of one researcher and class teacher | Mother-child F&V score (T1): 0.24; Mother-child excess score (T2): 0.28; Mother-child F&V score (T4): 0.24; Mother-child excess score (T4): 0.29; | Weak |
| 11 | ^12^Billon, et al. (2002)  France | Cohort | 1595 | 10-18 years | 35-48 years | 3-day food record | Absolute and relative breakfast energy intakes (BEI and RBEI) | Self-completed diary by parent and child | Father-son (BEI): 0.25; Father-daughter (BEI): 0.19; Mother-son (BEI): 0.26; Mother- daughter (BEI): 0.27; Father- son (RBEI): 0.23; Father- daughter (RBEI): 0.19; Mother-son (RBEI): 0.27; Mother-daughter (RBEI: 0.21 | Weak |
| 12 | ^13^Stafleu A. et al. (1994)  Netherlands | Cross-sectional | 291 | 18 years | 42-55 years | FFQ, 104 items | Fat, Fatty acids, cholesterol and energy | Self-reported | Weak correlations (0.13-0.27) were found between nutrient intake of the younger and middle generation. Pearson correlations: Energy (kJ): 0.22; Energy (kJ/kg body wt): 0.27; | Weak |
| 13 | ^14^Wroten, et al. (2012)  USA | Cross-sectional | 1300 | 5 years | 18-49 years | 24h recall | snack, sweet, fruit, vegetable | Interviewer administered | **Total:** Mother-Child (Sweet): 0.345; Mother-child (Snack): 0.340; Mother-child (Fruit): 0.359; Mother-child (Vegetable): 0.476; Mother-child (Energy): 0.483; **African American:** Mother-Child (Sweet): 0.369; Mother-child (Snack): 0.233; Mother-child (Fruit): 0.249; Mother-child (Vegetable): 0.394; Mother-child (Energy): 0.358. **Hispanics:** Mother-Child (Sweet): 0.291; Mother-child (Snack): 0.345; Mother-child (Fruit): 0.327; Mother-child (Vegetable): 0.441; Mother-child (Energy): 0.485. **White:** Mother-Child (Sweet): 0.358; Mother-child (Snack): 0.457; Mother-child (Fruit): 0.237; Mother-child (Vegetable): 0.497; Mother-child (Energy): 0.329. | Weak-to-moderate |
| 14 | ^15^Vauthier, et al. (1996)  France | Longitudinal survey | 1548 | 2-7 years | 18- 65 years | 3-day dietary record | Nutrient intake | Mixed: Self-recorded; for young children mother completed the diary with the help of children | Father – son (Energy): 0.35 Father – daughter (Energy): 0.33; Mother – son (Energy): 0.24 Mother – daughter (Energy): 0.26 Father – son (Protein): 0.36 Father – daughter (Protein): 0.36 Mother – son (Protein): 0.26 Mother – daughter (Protein): 0.31 Father – son (Fat): 0.39 Father – daughter (Fat): 0.28 Mother – son (Fat): 0.31 Mother – daughter (Fat): 0.40 Father – son (CHO): 0.37 Father – daughter (CHO): 0.26 Mother – son (CHO): 0.28 Mother – daughter (CHO): 0.35 | Weak-to-moderate |
| 15 | ^16^Mitchell, et al. (2003)  USA | Population-based study | 1364 | 16-18 years | 40-60 years | FFQ, 102 items | Total calories, total protein, total fat, and total CHO | Trained interviewers | Parent-offspring (Calories): 0.14; Parent-offspring (Total CHO): 0.05; Parent-offspring (% protein): 0.10; Parent-offspring (% fat): 0.09; Parent-offspring (saturated fat): 0.11 | Weak |
| 16 | ^17^Hebestreit, et al. (2017)  Eight European countries; Sweden, Germany, Hungary, Italy, Cyprus, Spain, Belgium, and Estonia | Cross-sectional | 4816 | 6-16 years | 30-54 years | 24h recall | Individual usual intakes of Nutrients and Food group | Mixed: Self-reported and Children < 11 years took their parents help | **Children-Mother resemblance (Shared meal)**: Children-Father (Shared meal): Sweet and Fat DP: OR 1.91; 95% CI 1.17- 3.13; Refined Cereals: OR 2.70; 95% CI 1.34 -5.45; Animal Products: OR 2.19; 95% CI 1.41 -3.40; Children-Father (Shared meal) OR 3.18; 95% CI 1.15; Sweet and Fat DP was highest among children if mother is in the same group, OR 2.78; 95% CI 1.84 -5.47; Refined Cereals: OR 1.99; 95% CI 0.98-4.08; Animal Products: OR 1.54; 95% CI 0.91-2.59: **Children-Mother (Soft drinks available)**: Child- Mother (Soft drinks available) in Sweet and Fat DP: OR 2.04; 95% CI 1.49-2.80; Refined Cereals: DP OR 2.48; 95% CI 1.43- 4.27; Animal Products DP: OR 2.16; 95% CI 1.59 - 2.92; Child- Father (Soft drinks available) in Sweet and Fat DP: OR 2.48; 95% CI 1.58-3.87; Refined Cereals: DP OR 2.05; 95% CI 1.22- 3.45; Animal Products DP: OR 2.48; 95% CI 1.62- 3.79 | Strong |
| 17 | ^18^Grimm, et al. (2004)  USA (including states in the East, West, South, Midwest, and Alaska) | Survey | 560 | 8-13 years | 18-49 years | Short Questions | soft drinks | Self-reported | Parent-child (soft drink): Children 7 years old- OR 4.41; 95% CI 2.92- 6.67; Child 13 years old: OR 2.88; 95% CI 1.76- 4.72; | Strong |
| 18 | ^19^Gibson, E.L., 1998 Europe (South London, England) | Cross-sectional | 9 | 9–11 years | 34-44 years | Mixed: FFQ (Mother) and 3-day diaries (Children) | Fruit and vegetable, confectionery intake. | Self-reported | Mother-child resemblance (Fruit): 0·077; SE B: 0.026; Beta: 0.30; t 2.94; p(t): 0.0046; mothers - children (vegetable) (r_s_ −0·02) or confectionery intake (r_s_ =0·11, NS) | Weak-to-Moderate |
| 19 | ^20^Hall, L., et al., 2011  New South Wales, Australia | cross-sectional | 184 | 5 - 12 years | 21-65 years | FFQ, 74 items | Nutrient intake | Mixed: Self-reported (Father) and Completed by mother (Child) | Father-child: Energy (kcal/d): 0.17 Protein (% energy): 0.11; Carbohydrate (% energy): 0.01; Fat (% energy): 0.07; Saturated fat (% energy): 0.25; Total fat (g/d): 0.10; Saturated fat (g/d): 0.13; Fiber (g/d): 0.20; Calcium (mg/d): 0.26; Iron (mg/d): 0.29; Vitamin C (mg/d): 0.34; β carotene (mg/d): 0.18; Sodium (mg/d): 0.17; Total fruit (g/d): 0.35; Fruit excluding juice (g/d): 0.40; Fruit juice (mL/d): 0.31; Total vegetable (g/d): 0.07; Total vegetable except French fries (g/d): 0.18; French fries (g/d): 0.15; Chocolate (g/d): 0.05 Chips (potato and non–potato-based): (g/d): 0.33; Cookies (g/d): 0.54; Ice cream (g/d): 0.20 | Moderate to strong |
| 20 | ^21^Dondero, et al. (2016)  USA | Survey | 2090 | 2-15 years | 18-49 years | 24h recall | Healthy Eating Index score (HEI). | Mixed: Mother: Self-reported for mother (interview administered); Proxy reported for aged <5; 6-11 aged assisted with interview | Mother's HEI and child's HEI resemblance: Children of Mexican immigrant mothers’ weaker correlations than children of Mexican American mothers: r = 0.16 and r = 0.28 for first and second-generation children, compared to r= 0.34 for third-generation children. By Child's generation status: First generation child: (β_1st_ = 0.14) relative to both second (β_2nd_ =0.27) and third-generation children (β_3rd_ =0.33), though the difference in slopes is significant only between the first and third generations. | weak |
| 21 | ^22^Fisher, et al., (2001)  USA | Cross-sectional | 368 | 5 years | 18-45 years | Mixed: FFQ (Mother); 24 hr recall (Child) | Energy, calcium, milk and sweetened beverage intake | Mother: Self-reported and reported for child | Mother-daughter (Milk): r=0.22; Mother-daughter (Soft drinks): r=0.17 | weak |
| 22 | ^23^Reinaerts, et al. (2007)  Netherlands | longitudinal study | 1739 | 4 -12 years | 28-49 years | Frequency method by pro-children questionnaire (Child); 10-item Q (Parent) | fruit, vegetables | Parent | Parent-child (fruit): r= 0.15; Parent-child (Vegetable): r= 0.07; | weak |
| 23 | ^24^Oliveria, et al., (1992)  USA | Cross-sectional | 261 | 3 and 5 years | 18-40 years | 3-day food diary | Individual usual intakes of Nutrients | Mixed: parent and adult personnel at day care centre | Father-child (Energy): 0.16; Protein: 0.34; CHO: 0.18; Total fat :0.15; Saturated fatty acid (SFA): 0.34; Mono saturated fatty acid (MSFA): 0.13; Poly saturated fatty acid (PSFA): 0.10; Cholesterol: 0.34; Sodium: 0.18; Potassium: 0.04; Calcium: 0.21 Mother-child (Energy): 0.20; Protein: 0.37; CHO: 0.31; Total fat: 0.32; SFA: 0.47; MSFA: 0.29; PSFA: 0.27;  Cholesterol: 0.41; Sodium: 0.21; Potassium: 0.12; Calcium: 0.30 | Weak-to-moderate |
| 24 | ^25^Raynor, et al. (2011)  USA | cross-sectional | 270 | ≥ 8 years | 32-44 years | 3-day dietary record | Individual usual intakes Food group | Mixed: parent and adult personnel at day care centre | Parent-child intake (Fruit): r = 0.226; Parent-child intake (Vegetable): r = 0.298; Parent-child intake (Low Fat Dairy): r = 0.447; Parent-child intake (Snack Foods): r = 0.238; Parent-child intake (Sweetened Bev): r = 0.222; | Weak-to-moderate |
| 25 | ^26^Cameron, et al. (2011)  Australia | cross-sectional | 608 | 5-12 years | 18-45 years | FFQ | Healthy eating behaviours and Unhealthy eating behaviours | Mother | Mother with healthy eating had children with healthy eater; cluster membership: 26 and 15 (Concordance is strong) | Strong |
| 26 | ^27^Fisher, et al. (2002)  USA | cross-sectional | 382 | 5 years | 35 years | Mixed: FFQ (Mother); daughters' (24h recall) | Fruits and vegetables | Mother (self-reported); Child (Mother); interviewer administered | Parent-daughter resemblance: Parent-daughter (F & V): 0.23 | weak |
| 27 | ^28^Longbottom et al.,2002 | Cohort | 36 | 5-9 years | 18-45 years | 4-day dietary record | Individual usual intakes of Food groups | Mother | Positive, significant correlations between children and mothers were found for median densities of bread (r = 0.360, P < 0.05), fruit (r = 0.735, P < 0.001) and potatoes (r = 0.572, P < 0.001) and also for chips (r = 0.651, P < 0.001) and chocolate confectionery (r = 0.368, P < 0.05) | Moderate to strong |
| 28 | ^29^Best, J.R., et al., 2016 | Cross-sectional | 269 | 7–12 years | 27–66 years | 4-day dietary record | Individual usual intakes of Food groups | Self-reported | Nutrient-poor, energy-dense foods: PC 0.47; Fruits and vegetables: PC 0.26 | Weak -moderate |
| 29 | ^30^Perusse, et al., (1988)  Canada, Quebec | cross-sectional | 1597 | 8-18 years | 18-59 years | 3-day dietary record | Individual usual intakes of Nutrient | Self-reported | Parent-child (r for ICC): Energy intake (kcal): 0.27; Energy intake (kcal/kg’/d’): 0.38; Carbohydrate: 0.30; Fat (g): 0.26; Protein (g): 0.30; CHO (%): 0.38; Fat (%):0.38; Protein (%): 0.45; Linoleic acid: SFA ratio: 0.49 | Weak -moderate |
| 30 | ^31^Vollmer, et al., (2015)  USA | Cross sectional | 150 | 3-5 years | 30-45 years | Mixed: 24h recall and Healthy Eating Index-2010 | Individual usual intakes of Nutrients and Food group | Self-reported (Father) | Father-child: overall diet quality (b=.39; P<0.0001), and weekday (b=.27; P=0.002) and weekend (b=.62; P=0.001); Father-child (β–SE): Overall diet quality: 0.40 ± 0.09; Adequacy: .35 ± .08; Total fruit: .18±.08; Whole fruit: .20±.0; Total vegetables: .29±.06; Greens/beans: .27±.06; Whole grains: .28±.08; Dairy: .00±.07; Total protein: .09±.13; Plant/seafood protein: .23±.09; Fatty acids ratio: .19±.08; Moderation: .34±.08; Refined grains: .32±.07; Sodium: .24±.08; Empty calories: .24±.07 | Weak -moderate |
| 31 | ^32^Galloway, et al. (2005)  USA | longitudinal study | 346 | 7 & 9 years | 30-49 years | Mixed: FFQ (ay 7 years); 24-h recall (at 9 years) | fruit, vegetable, micro-nutrient, and fiber intakes. Picky eating. | Mother | Mother-daughter (F & V): r=0.36; Mother-daughter (Soft drinks): r=0.17 | moderate |
| 32 | ^33^Laskarzewseki et al. (1980)  USA | Cross-sectional | 588 | 6-19 years | 20-69 years | 24h recall | Nutrient intake | Mixed: Mother: Self-reported for mother; Proxy reported for aged <5; 6-11 aged assisted with interview; Child (self-reported) | Parent-child: Total CHO (Pearson) 0.223; Total CHO (Spearman): 0.28 Cholesterol (Pearson): 0.056 Cholesterol (Spearman): 0.004 Saturated Fat (Pearson) 0.224 Saturated Fat (Spearman): 0.154 PUFA (Pearson): 0.164 PUFA (Spearman): 0.191 Calories (Pearson): 0.250 Calories (Spearman): 0.241 | weak |
| 33 | ^34^Johnson, et al. (2011)  UK | Cross-sectional | 513 | 11 years old | 37-46 years | FFQ | Core foods and Non-core foods | Mother | Maternal food intake and child's core food intake: 0.14; Non-core food: 0.08; Parental food intake and core food intake: 0.07; and Non-core food: 0.03 |  |
| 34 | ^35^Hannon, et al. 2003  USA | cross-sectional | 282 | 5-17 years | 36-69 years | Fat-and-fiber-related diet behaviour questionnaire (FFB); 36 items | high-fat foods and fruit and vegetable intake | Mother or Family food preparer (FFP) | Parents' high intake of F & V predicts child’s and adolescents' high intake of F & V= 4.22 and 4.54; High fat foods may predict high fat foods of children and adolescent = 3.05 and 1.97; (Note: Predicted least squares means were derived from a multivariate mixed model, with RO specified as a random effect, and FFP fat intake specified as a fixed effect) | Strong |
| 35 | ^36^Murrin, et al. (2015)  UK, Ireland | longitudinal study | 1430 | 5 years | -- | FFQ, 149 food items and FFQ (children) | Individual usual intakes of Food groups | Mother | The children’s “pasta & vegetable” pattern was positively correlated with “healthy patterns” in mothers (r = 0.195, p < 0.01) and fathers (r = 0.250, p < 0.01). The children’s “junk” food pattern was correlated with the “processed” pattern in mothers (r = 0.245, p < 0.01) and fathers (r = 0.257, p < 0.01). | Weak |
| 36 | ^37^Lipsky, L. M., et al. 2019 | secondary data analysis | 272 | 10-14 years | -- | 3 d food record | Healthy Eating Index-2005 (HEI-2005); whole plant food density (WPFD) |  | Parent-child WPFD resemblance was stronger in the intervention (β [SE]=.30 ± .06) vs control families (β ± [SE]=.12 ± .05). Parent-child HEI-2005 resemblance was similar over time by treatment assignment, whereas parent-child WPFD resemblance increased over time for families in the intervention group (three-way interaction term β±[SE]=.03 ± .01). Parent-child resemblance (HEI-2005: Intervention group): (β± [SE]=.25 ± .06) and (HEI-2005: Control group): (β± [SE]=.28 ± .06). Parent-child resemblance (WPFD: Intervention group): (β± [SE]=.30 ± .06) and (WPFD: Control group): (β± [SE]=.12 ± .05) | Moderate |
| 37 | ^38^Stanton, et al. 2003  USA | Cross-sectional survey | 808 | 12-15 years | Mothers' age: 27-71 years | FFQ; 35 food items | Fat, fibre intake | Mother | Significant dietary fat concordance rates were indicated. Mother-children: r = .22; Pearson correlations for mother-daughter dyad: r=.30; mother-son dyad: r=.11; white mother-child dyads: r=.23; Africa-American dyad: .18 | low to moderate |
| 38 | ^39^Patterson, et al. (1988)  USA | cross-sectional | 589 | 2-10 years |  | Mixed: 24h recall, 3-day food record, FFQ | Whole diet |  | Mothers’ diets were more highly correlated with the children’s diets than fathers. The diets of the younger children, but not the older children, were related to their parents’ diets | Moderate |
| 39 | ^40^Vivarini, et al (2019)  Australia | Cohort | 2573 | 11-12 years | Parent age: 43.9 ± 5.6; Father; 14.1% of parent population | Food stop procedure, 15 mins station offered roughly midway through the 3.5-hour pre-set circuit at the CheckPoint’s Main Assessment Centre visits. | Energy and nutrient intake: Food weight grams, Energy (kJ), Protein (g), Saturated fat (g), Sodium (mg), Sugar (g), Carbohydrate (g), Total fat (g) | Self-reported | Food (g): 0.14 (0.07 to 0.20); Energy (kJ): 0.19 0.12 to 0.26; Protein (g): 0.17 0.09 to 0.23; Saturated fat (g): 0.10 0.02 to 0.17; Sodium (mg): 0.08 0.01 to 0.15; Sugar (g): 0.14 0.07 to 0.20; Carbohydrates (g): 0.22 0.15 to 0.28; Total fat (g): 0.13 0.06 to 0.20 | Weak-to-moderate |
| 40 | ^41^Elfhag, et al. (2008)  Sweden | Cohort | 4707 | 11- 12 years | mother's age: 40.0 ± 4.3 and Father's age: 42.4 ± 4.2 | FFQ | Food groups | Self-reported | Parent-Child food intake correlations: Fruit: Father-daughter: 0.20; Mother-daughter : 0.27; Mother- son : 0.15; Father- son : 0.15 Vegetables: Mother-daughter: 0.19; Father- daughter: 0.21; Mother–son: 0.21; Father–son: 0.23 Sweets: Mother-daughter: 0.22; Father-daughter: 0.25; Mother-son: 0.21; Father-son: 0.07  Soft drinks: Mother- daughter: 0.27; Father- daughter: 0.25; Mother-son: 0.31; Father-son: 0.25 | Weak-to-moderate |
| 41 | ^42^Bere, et al. 2004  Norway | Survey | 3597 | 11-12 years | Avg parents was 40.0 years | FFQ | Fruits and Vegetables intake | Self-reported | The correlation between the parent-child fruit and vegetable intake was 0.23 | Weak |
| 42 | ^43^Rossow, et al. (1994)  Norway | National health Survey | 984 | 16-20 years | 82% of all families with children were two parent families | Short questions | fatty dietary intake (milk and butter/margarine) | Self-reported | Mother-child: 0.47; Father-child: 0.42 Children were more likely to have a low-fat diet if their parents did so: mother’s low: OR: 5.00 (2.97, 8.41); father’s low: OR: 4.44 (2.56, 7.85) | Moderate |
| 43 | ^44^Adelekan, et al. (1997)  Nigeria | Cross-sectional study | 216 | 3–5 years | Mother's mean age: 31.0; Rural residents | 3 consecutive days of 24-hour recalls | Energy, Total Fat, Protein, Iron | Mother | Mother-child pair: energy (kJ): 0.39 protein (g): 0.07; total fat (g): 0.03; iron (mg): 0.55 | Weak-to-moderate |
| 44 | ^45^Feunekes et al. (1998)  Netherlands | Survey | 553 | 15 years | Mothers age: 42 ± 4; Fathers age: 44 ± 5 | FFQ | Energy, Fats (Total Fat, Saturated, Monounsaturated, Polyunsaturated and Cholesterol intakes | Self-reported | Mother-child: Energy (MJ): 0.19; fat (% energy): 0.19; Saturated fat (% energy): 0.23; MUFA (% energy): 0.20; PUFA (% energy): 0.38; Cholesterol (mg/MJ): 0.00 Father-child: Energy (MJ): 0.13; Energy (MJ/kg): 0.10; Total fat (% energy): 0.18; Saturated fat (% energy): 0.24 MUFA (% energy): 0.26; PUFA (% energy): 0.16; Cholesterol (mg/MJ): 0.22; Energy (MJ/kg): 0.22 | Weak-to-moderate |
| 45 | ^46^Runyan et al. (2003)  USA |  | 144 | 11–14 y (12.8±0.8 y) | mother aged 33- 51 years old with mean age as 42.4±4.2 | 3-day food records with a calcium intake survey | Calcium intake | Self-reported | Mother-Daughter (Calcium): 0.35; Mother (Daughter)-Grandmother (Mother) (Calcium): 0.33* | Moderate |
| 46 | ^47^Park et al. (2004)  South Korea | Cross-sectional | 491 | 11–19 years | Average parental age was 43.6 (32–57) years for fathers and 40.9 (30–53) years for mothers | FFQ | Energy intake, Carbohydrate, Protein, Fat Saturated, fat Dietary cholesterol | Self-reported | Father-son: Energy: −0.03; CHO (% energy): 0.12; Protein (% energy): 0.02; Fat (% energy): 0.20; Saturated fat: 0.04; Cholesterol: −0.06 Father-daughter: Energy: 0.20; CHO (% energy): −0.10; Protein (% energy): 0.07; Fat (% energy): −0.01; Saturated fat: 0.02; Cholesterol: 0.03 Mother-son: Energy: 0.10; CHO (% energy): 0.23; Protein (% energy): 0.23; Saturated fat: 0.28; Cholesterol: 0.27 Mother-daughter: Energy: 0.27; CHO (% energy): 0.17; Protein (% energy): 0.31; Fat (% energy): 0.09; Saturated fat: 0.25; Cholesterol: 0.14 | Weak |
| 47 | ^48^da Veiga, et al (2006)  Brazil | Cross-sectional | 1420 | 12–18 y |  | FFQ | Food groups | Self-reported | Rice (1 soupspoon): Father-daughter: 0.36; Father-son: 0.49; Mother daughter: 0.46; Mother-son: 0.36 Milk products (1 glass or slice or unit): Father-daughter:0.51; Father-son: 0.63; Mother-daughter: 0.60; Mother-son: 0.58 Meat (4–6 oz): Father-daughter: 0.53; Father-son: 0.63; Mother-daughter: 0.56; Mother-son: 0.58 Soda (1 glass): Father-daughter: 0.25; Father-son: 0.42; Mother-daughter: 0.39; Mother-son: 0.39 | Moderate |
| 48 | ^49^López- Alvarenga et al. (2007)  Mexico |  | 1506 | 8–12 y | Father age: 31-51 years and Mother's age: 30-46 years in both schools. | Questionnaire 43 questions | Food groups | Parent | child- parent food preference: Yogurt, Oaxaca cheese and yellow cheese: 0.75; Skimmed milk, skimmed yogurt, and diet soda: 0.67; Omelette, bread and beans: 0.80; Eggs, pork, butter, fried potatoes, tacos, soups, sandwiches and pizza: 0.77; Fish: 0.79 Sausages, beef, poultry and oat: 0.71; French fries, sweet bread, cakes and sweetened cereals: 0.81; VF: 0.74; Sweets and soft drinks: 0.74; Whole milk: 0.50 | Strong |
| 49 | ^50^Papas et al, (2009)  USA | longitudinal study | 218 | 13 months old | Age: 14-19 years; primiparous, low-income, African American mothers and their toddlers | 73-item feeding checklist; maternal diet variety using youth Adolescent Food Frequency Questionnaire (YAQ). | Food groups | Mother | fruit: 0.26; vegetables: 0.40; snacks/disserts: 0.50; meats: 0.29; diary: 0.18; soda: 0.25 | Weak-to-moderate |
| 50 | ^51^Rozin, et al, 1991  USA | Survey (Student class project) | 118 | 17-19 yrs of age | Mother's mean age: 46.0 (SD=3.7); Father's mean age: 48.92 (SD=4.9) | Questionnaire | Food groups | Self-reported | The overall correlation for food domain among congruent parents with child was 0.18 as opposed to 0.11 for non-congruent parents. | Weak |
| 51 | ^52^Hart, et al, 2010  USA | Cross-sectional study | 98 | 6-18 months | Mean age of mothers was 24.2 (5.5) years and mostly are African American mothers | WIC Food Frequency Questionnaire (FFQ) | Fruit, vegetable, and snack intake | Interviewer administered | Infants’ and toddlers’ fruit (r = 0.54, P < 0.001), vegetable (r = 0.42, P < 0.001) and snack food (r = 0.37, P < 0.001) intake were significantly associated with maternal intake of each of these foods, respectively. | Moderate |
| 52 | ^53^Kunaratnam, K. et al, 2018  Australia | Cross-sectional study | 486 | 2 years of age | Mothers’ mean age 26 years (SD 6 months) and age ranged from ≥16 to ≤47 years | Short FFQ from the New South Wales Child Health Survey for child & Mothers’ own dietary questions sourced from the New South Wales Health Survey Program in New South Wales. | Intake of vegetables, fruit, water, milk, soft drinks, sugary drinks, processed meats, fast food, hot chips, confectionery, salty snacks, and sweet snacks | Interviewer administered | Significant positive correlations for all dietary variables except milk, r=0.08, (p>0.05), fruit juice intake, r= 0.27 with the largest association for fast foods, r 0·52 (P<0·001) and moderate associations for water, r=0.37, fruit, r=0.48, vegetables, r=0.40, sugary drinks, r=0.37, hot chips=0.37, soft drink r= 0.30 and processed meat, r=0.34 (Pearson’s r varied from 0·27 to 0·48, P<0·001) | Weak to Strong |
| 53 | ^54^Prichard, I, 2012  Australia | Cross-sectional study | 112 | 17-25 years (mean-19.21, SD=1.67) |  | modified version of Campbell et al.’s (2006) Food Frequency Questionnaire (FFQ) and Dutch Eating Behaviour Questionnaire (DEBQ) | Vegetable and energy-dense foods (EDFs) including high energy (non-dairy) fluids, sweet snacks, and savoury snacks. | Self-administered/self-reported | Mothers’ EDF consumption (b = .71, p < .001) had more impact upon daughters’ food intake than the daughters’ level of restrained eating (b = -.24, p < .001) and mothers’ vegetable intake (b = .14, p <.05). vegetable intake. Mothers’ vegetable intake (b = .56, p < .001) with daughters’ vegetable intake | Strong |
| 54 | ^55^Tada, Y, 2015  Japan | Cross-sectional study | 664 | 10-12 years (Mean age-10·9 (SD 0·7) years) | Mean age for mothers and children was 42·9 (SD 3·9) years. | Mother: Validated self-administered brief diet history questionnaire (BDHQ); Child: modified version of the BDHQ (BDHQ-10y) | Vegetable intakes | Mother: Self-administered; Child: self-administered and mother reported for child as well. | Mothers’ vegetable intake was significantly correlated with children’s vegetable intake. A positive linear relationship was found between mothers’ and children’s vegetable intake even after adjustment for considerable covariates (P<0·001). |  |
| 55 | ^56^Broek, N.Van, 2020  Netherlands | Cross-sectional study | 1441 | 10.0 - 14.8 years and Mean age- 12.9 (SD=0.7) | 29.8 - 57.3 years and mean age of the mothers was 44.7 years (SD= 4.2) | Validated Dutch Food frequency questionnaire (FFQ). | Intake of SSBs, sweet snacks, savory snacks, and fruit and vegetables | Both: Self-administered | The correlation coefficients indicate that for all food types, adolescents’ intake of foods obtained from home was related to their mothers’ total food intake, and their mothers’ intake in the presence of their child. SSBs: 0.13; Sweet snacks: 0.18; savory snacks: 0.14; Fruits and vegetables: 0.21. | Weak |
| 56 | ^57^Thorsdottir, I, 2006  Iceland | Cross-sectional study | 375 | 6 years |  | Parent: food frequency questionnaire (FFQ); Children: 3-day dietary record | Fruit and vegetables intake, vitamin C and Beta-carotene intake. | Parents: Self-administered; Child: Parent reported | A positive correlation was seen in serum vitamin C concentration for Mother: r=0.454, p<0.005 and Father: r=0.529, p<0.005. Serum b-carotene concentration Mother, r=0.385, p<0.0) and Father, r=0.419, p<0.01) between family members. Family members’ intake of fresh fruit, fruit juice and green leafy vegetables was positively related (r=0.227, p<0.05 and r=0.313, p<0.01 between children and their mother and father. | low to moderate |
| 57 | ^58^Laster, LER, 2013  USA | Cross-sectional study | 354 | 3-5 years old, mean=3.5 (SD=1.03) | Maternal age (y), mean=32.2 (SD=4.7) | Healthy Eating Index-2005 | fruits, vegetables, whole grains, meat and beans, sodium, saturated fat, and energy from solid fat and added sugars | Mother | Child diet quality was correlated with maternal diet quality (r=0.44; P<0.0001) | Moderate |
| 58 | ^59^Vanhala, ML, 2010  Finland | Cross-sectional comparative study | 307 | 8-year-old | Biological mothers (n = 105) lived with their children. 88 % biological fathers lived with their children on weekdays. | Food frequency questionnaire (FFQ) | Intake of fruit, berries, and vegetables (FBV) | Child: Parent reported; Parent; Self-administered | Parental intake of FBV was significantly associated with the intake of their children (Spearman correlation for mothers 0.572 and 0.437 for mothers). Normal-weight children and parents ate FBV more frequently than overweight children. In the multiple linear regression analysis, mother’s (b = 0.476, P £ 0.001) and father’s consumption of FBV (b = 0.347, P = 0.001) and child’s preference for eating vegetables (b = 0.259, P = 0.002) were positively associated with the child’s consumption of FBV. In overweight children, parent’s consumption of FBV was the only predictor of the offspring’s consumption of FBV (P = 0.002). | Moderate |
| 59 | ^60^Harris, TS. Et al, 2015  USA | Cross-sectional study | 204 | 3-13 years | Age: 23-68 years; African American males (i.e., biological father, stepfather, and grandfather) | Comprehensive Feeding Practices Questionnaire (CFPQ) | Intake of fruits, vegetables, and sugar sweetened beverages | Father: Self-administered; Child: Father reported | Father and child association: Vegetable; r= 0.67; Fruit: r= 0.51; SSBs: r= 0.67 | Strong |
| 60 | ^61^Williams, A., 2018  Australia | Randomised controlled trial | 183 | 5–12 years | Fathers: Intervention group- mean age= 39.8 (SD=5.0) and control group: 40.9 (SD=5.6). Having Overweight or obese status [body mass index (BMI) 25–40 kg m–2] | Food frequency questionnaire (FFQ); Father: Australian Eating Survey (AES) and Child: Australian Child and Adolescent Eating Survey (ACAES) | Macronutrient, micronutrient, core foods, food groups | Father: Self-administered; Child: Mother reported | Father-child correlations (r):  CHO (% energy): 0.37; Protein (% energy): 0.28; Fat (% energy): 0.35; Saturated fat (%): 0.30; Core food: 0.47; EDNP foods (%): 0.47; Vegetable (%): 0.53; Fruit (%): 0.37; Meat (%): 0.05; Vegetarian protein (%): 0.19; Grain (%): 0.20; Dairy (%): 0.78; SSBs (%): 0.57; Prepacked snacks (%): 0.39; Confectionary (%): 0.21; Baked products (%): 0.28; Takeaway (%): 0.29; Condiments (%): 0.35; Fatty meats (%): 0.49; Breakfast cereal (%): 0.09; Meals with vegetables (%): 0.34; Meals without vegetables (%): 0.59 | Weak to Strong |
| 61 | ^62^Zuercher, JL., 2011  USA | Cross-sectional study | 4101 | 2-18 years |  | 24hour food recall and a second telephone recall collected three to ten days after the first recall | Food group (total grains, meat, fruit, vegetable, milk) and several nutrients (protein, saturated fat, dietary fiber, cholesterol, calcium, iron, folate, vitamin C, vitamin A and vitamin B12) | Parent: self-administered; Child (<6 years): Parent reported; Child (> 6 years): self-administered | Associations between food group/nutrient densities were significant but weak to moderate. | Weak to Moderate |

**Supplementary table 3.1: Summary of the main characteristics and findings of the 02 articles that reported parent-child resemblance in dietary intake since 2021 to 2022**

| **Ref no** | **Authors (year), Country** | **Study type** | **Sample size** | **Child age; mean (median/ SD/range)** | **Parent’s details** | **Dietary**  **assessment** | **Dietary variables** | **Who Reported dietary intake** | **Main Findings** | **Reported resemblance (Overall)** |
| --- | --- | --- | --- | --- | --- | --- | --- | --- | --- | --- |
| 1 | ^63^ Hosseini-Esfahani F., et al. (2022)  Tehran, Iran | Cohort | 3634 | 9-20+ years | 38-68 years | FFQ, 168 items | Food groups, nutrients, and Whole diet | Self-reported | Dietary quality and food group intakes in mother-offspring dyads (mother-son: 0.37, mother-daughter: 0.44.  Father-offspring dyads (father-son: 0.34, father-daughter: 0.25). | Weak to moderate |
| 2 | ^64^Mirmiran, P., et al. (2022)  Tehran, Iran | Cohort | 4685 | 9-20+ years | 38-68 years | FFQ, 168 items | Energy and nutrient intakes | Self-reported | Parent–offspring (r = 0.11–0.33); Parent–offspring correlations were stronger for the intake of healthy (r = 0.33) than unhealthy (r = 0.10) foods.  **Total energy (Kcal/day**), r:  Father-son (living with their parents): 0.11  Father-daughter (living with their parents): 0.07  Father-son (living independent of their parents): -0.04  Father-daughter (living independent of their parents): 0.12  **Carbohydrate**:  Father-son (living with their parents): 0.08  Father-daughter (living with their parents): 0.14  Father-son (living independent of their parents): -0.005  Father-daughter (living independent of their parents): 0.17  **Protein**:  Father-son (living with their parents): 0.15  Father-daughter (living with their parents): 021  Father-son (living independent of their parents): -0.03  Father-daughter (living independent of their parents): 0.13  **Total energy (Kcal/day**), r:  Mother-son (living with their parents): 0.11  Mother -daughter (living with their parents): 0.19  Mother -son (living independent of their parents): -0.01  Mother -daughter (living independent of their parents): 0.12  **Carbohydrate**:  Mother -son (living with their parents): 0.18  Mother-daughter (living with their parents): 0.36  Mother -son (living independent of their parents): -0.02  Mother -daughter (living independent of their parents): 0.17  **Protein**:  Mother -son (living with their parents): 0.14  Mother -daughter (living with their parents): 0.35  Mother -son (living independent of their parents): 0.02  Mother -daughter (living independent of their parents): 0.13 | Weak to moderate |

| 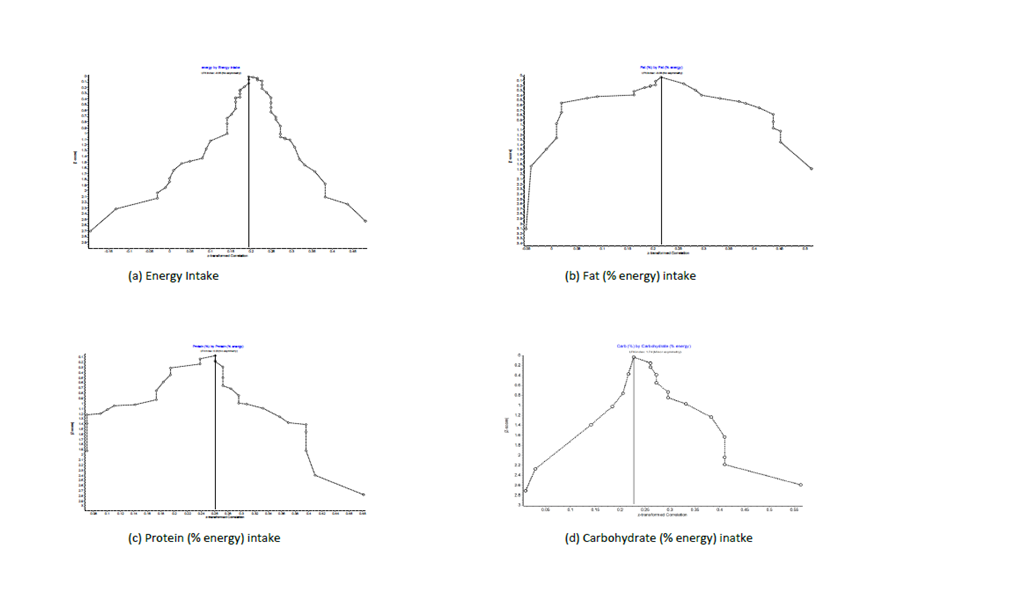 |
| --- |
| 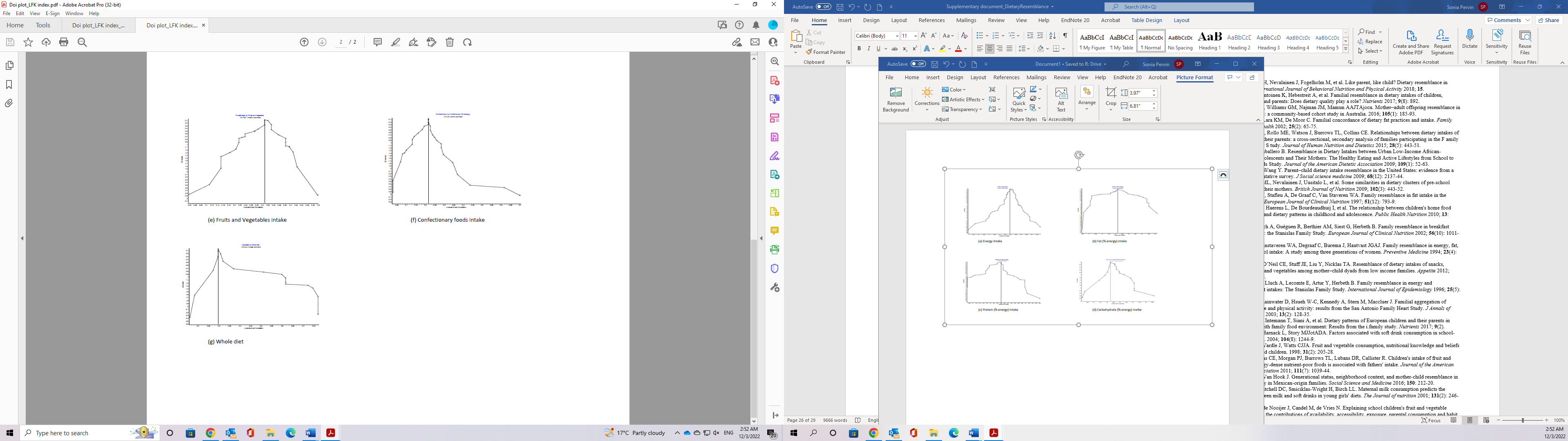 |

**Supplementary Figure 1:** **Publication bias using Doi plot and Luis Furuya-Kanamori (LFK) index of a) Energy intake; b) Fat (% energy) intake; c) Protein (% energy) intake; d) Carbohydrate (% energy) intake; e) Fruits and vegetable intake; f) Confectionary foods intake, and g) Whole diet.**

**
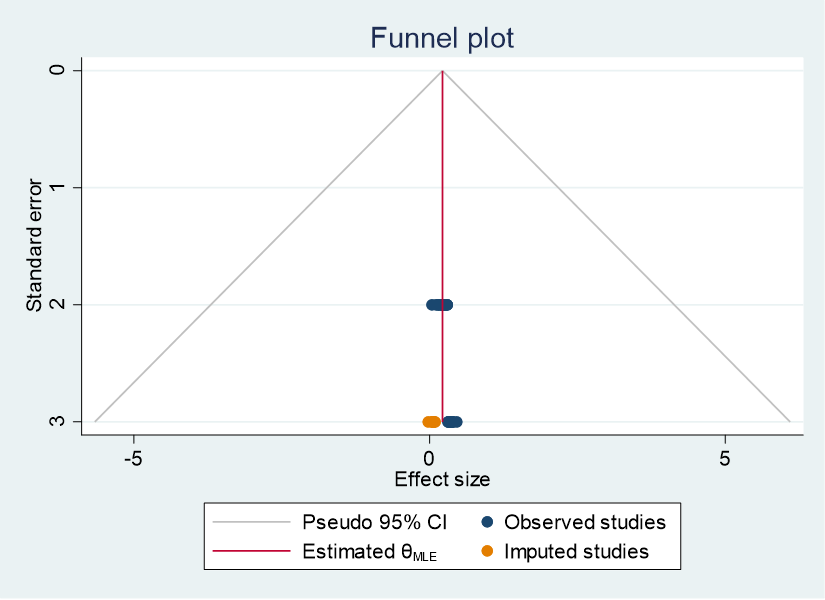
**

1. Fruits and vegetable intakes

**
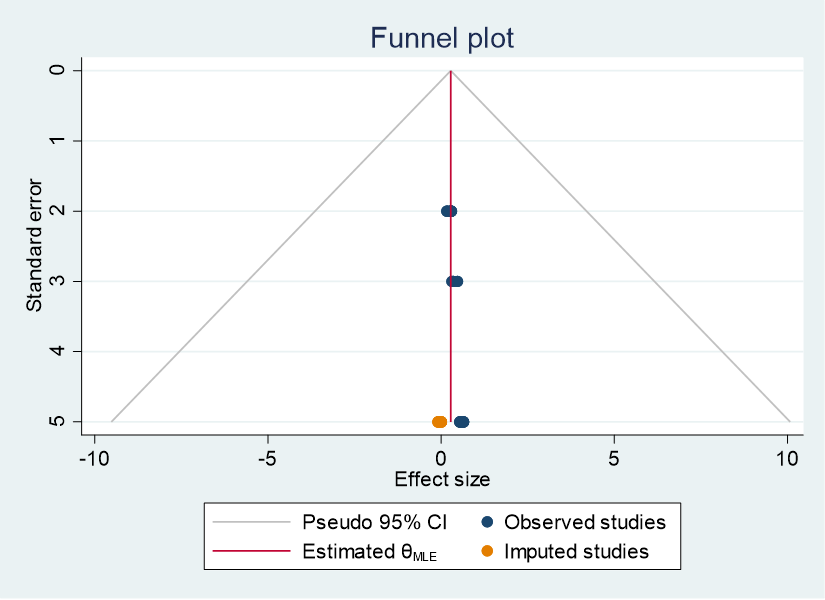
**

1. Whole diet

**Supplementary Figure 2: Depiction of publication bias in the trim and fill analysis for (a) Fruits and vegetable intakes [r: 0.22 (95% CI: -0.60, 1.09]; and (b) Whole diet: [r: 0.28 (95% CI: -0.92, 1.47].**

**Supplementary table 4: Summary table for sensitivity analysis of pooled estimation excluding articles of poor quality**

| **Study or Subgroup** | **Subgroup statistics** | **Subgroup correlation (95% CI)** | **% Weight** | **Overall Pooled correlation and statistics** |
| --- | --- | --- | --- | --- |
| **Energy (kcl/d)** | | | | |
| Parent-child Pairs | Q=49.40, p=0.00, I^2^=78% | 0.22 (0.17, 0.26) | 20.7 | 0.19 (0.15, 0.23); Q=612.48, p=0.00, I^2^=93% |
| Father-child Pairs | Q=418.46, p=0.00, I^2^=96% | 0.20 (0.10, 0.29) | 39.8 |  |
| Mother-child Pairs | Q=131.40, p=0.00, I^2^=78% | 0.18 (0.13, 0.23) | 39.5 |  |
| **Fat (% energy)** | | | | |
| Parent-child Pairs | Q=57.40, p=0.00, I^2^=97% | 0.22 (0.08, 0.36) | 14.9 | 0.31 (0.25, 0.37); Q=714.63, p=0.00, I^2^=97% |
| Father-child Pairs | Q=309.35, p=0.00, I^2^=97% | 0.31 (0.22, 0.40) | 42.8 |  |
| Mother-child Pairs | Q=270.53, p=0.00, I^2^=97% | 0.33 (0.24, 0.42) | 42.3 |  |
| **Protein (% energy)** | | | | |
| Parent-child Pairs | Q=76.86, p=0.00, I^2^=91% | 0.24 (0.16, 0.33) | 24.1 | 0.25 (0.20, 0.29); Q=372.26, p=0.00, I^2^=93% |
| Father-child Pairs | Q=241.39, p=0.00, I^2^=96% | 0.24 (0.14, 0.33) | 38.4 |  |
| Mother-child Pairs | Q=50.27, p=0.00, I^2^=84% | 0.25 (0.21, 0.30) | 37.5 |  |
| **Carbohydrate (% energy)** | | | | |
| Parent-child Pairs | Q=14.98, p=0.00, I^2^=87% | 0.25 (0.18, 0.32) | 23.9 | 0.25 (0.20, 0.30); Q=184.98, p=0.00, I^2^=91% |
| Father-child Pairs | Q=95.79, p=0.00, I^2^=93% | 0.22 (0.12, 0.31) | 42.0 |  |
| Mother-child Pairs | Q=51.55, p=0.00, I^2^=88% | 0.28 (0.20, 0.36) | 34.1 |  |
| **Fruits and Vegetables** | | | | |
| Parent-child Pairs | Q=28.93, p=0.00, I^2^=90% | 0.32 (0.12, 0.49) | 15.1 | 0.30 (0.25, 0.35); Q=291.13, p=0.00, I^2^=93% |
| Father-child Pairs | Q=138.52, p=0.00, I^2^=97% | 0.29 (0.18, 0.40) | 36.7 |  |
| Mother-child Pairs | Q=120.67, p=0.00, I^2^=92% | 0.30 (0.23, 0.36) | 48.2 |  |
| **Confectionery foods** | | | | |
| Parent-child Pairs | Q=9.37, p=0.01, I^2^=79% | 0.17 (0.10, 0.23) | 12.2 | 0.20 (0.16, 0.24); Q=323.00, p=0.00, I^2^=93% |
| Father-child Pairs | Q=176.07, p=0.00, I^2^=97% | 0.18 (0.09, 0.27) | 33.3 |  |
| Mother-child Pairs | Q=125.88, p=0.00, I^2^=90% | 0.21 (0.17, 0.26) | 54.5 |  |
| **Whole diet** |  |  |  |  |
| Parent-child Pairs | -- | -- | -- | 0.44 (0.33, 0.55); Q=289.48, p=0.00, I^2^=96% |
| Father-child Pairs | Q=0.09, p=0.99, I^2^=0.00% | 0.55 (0.51, 0.58) | 19.1 |  |
| Mother-child Pairs | Q=256.74, p=0.00, I^2^=97% | 0.42 (0.33, 0.55) | 80.9 |  |

**
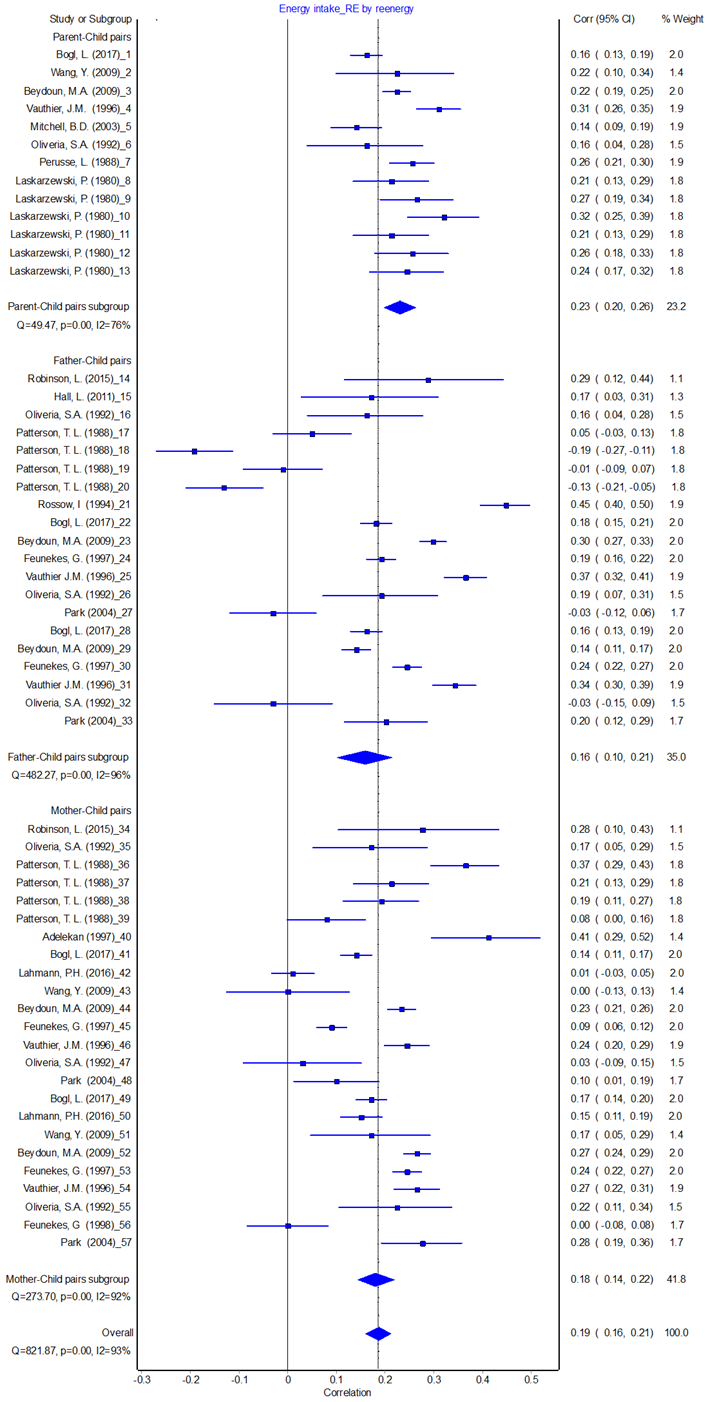
**

**Supplementary Figure 3: Sensitivity analysis of pooled estimation of energy intake between parent and child using random effect model.**

**
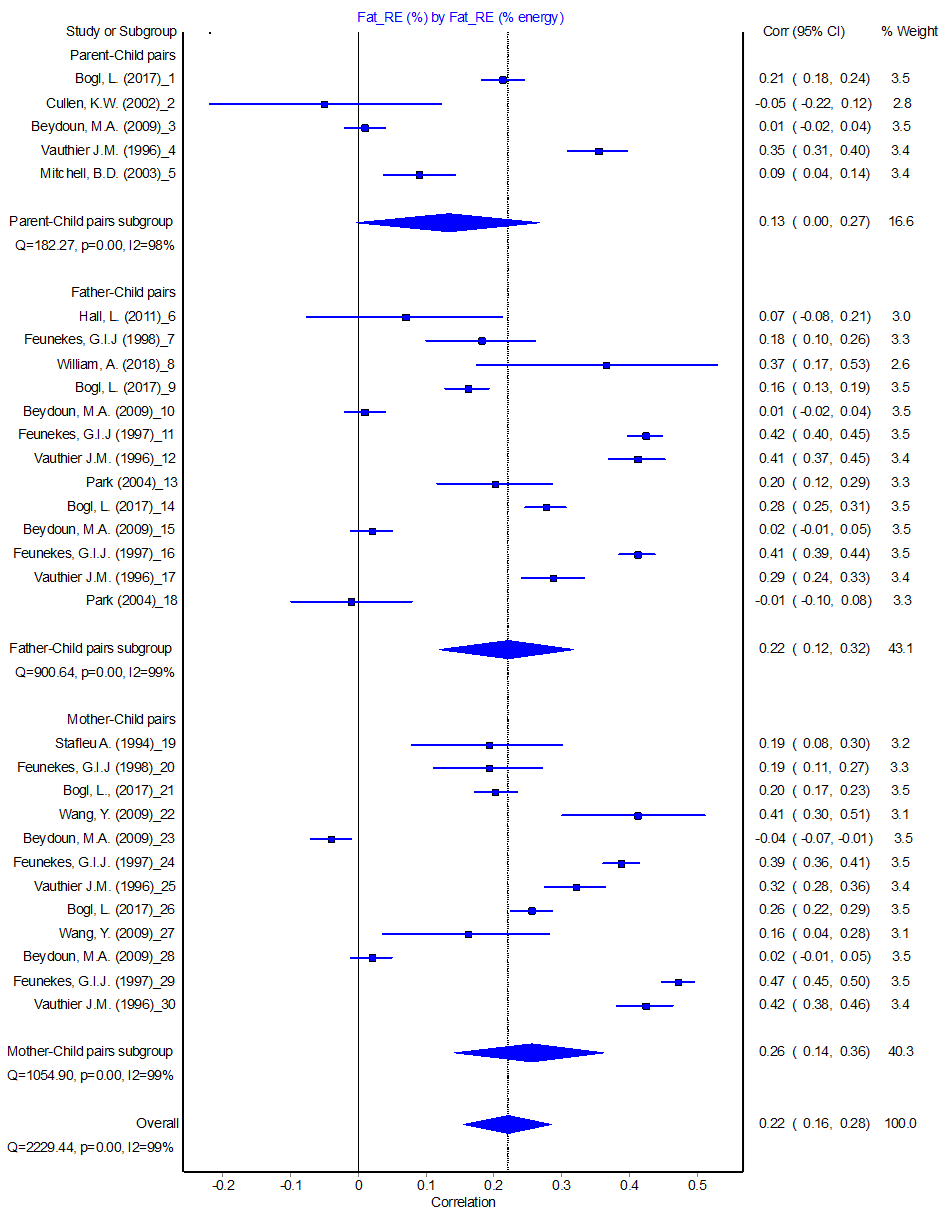
**

**Supplementary Figure 4: Sensitivity analysis of pooled estimation of fat (% energy) intake between parent and child using random effect model.**

**
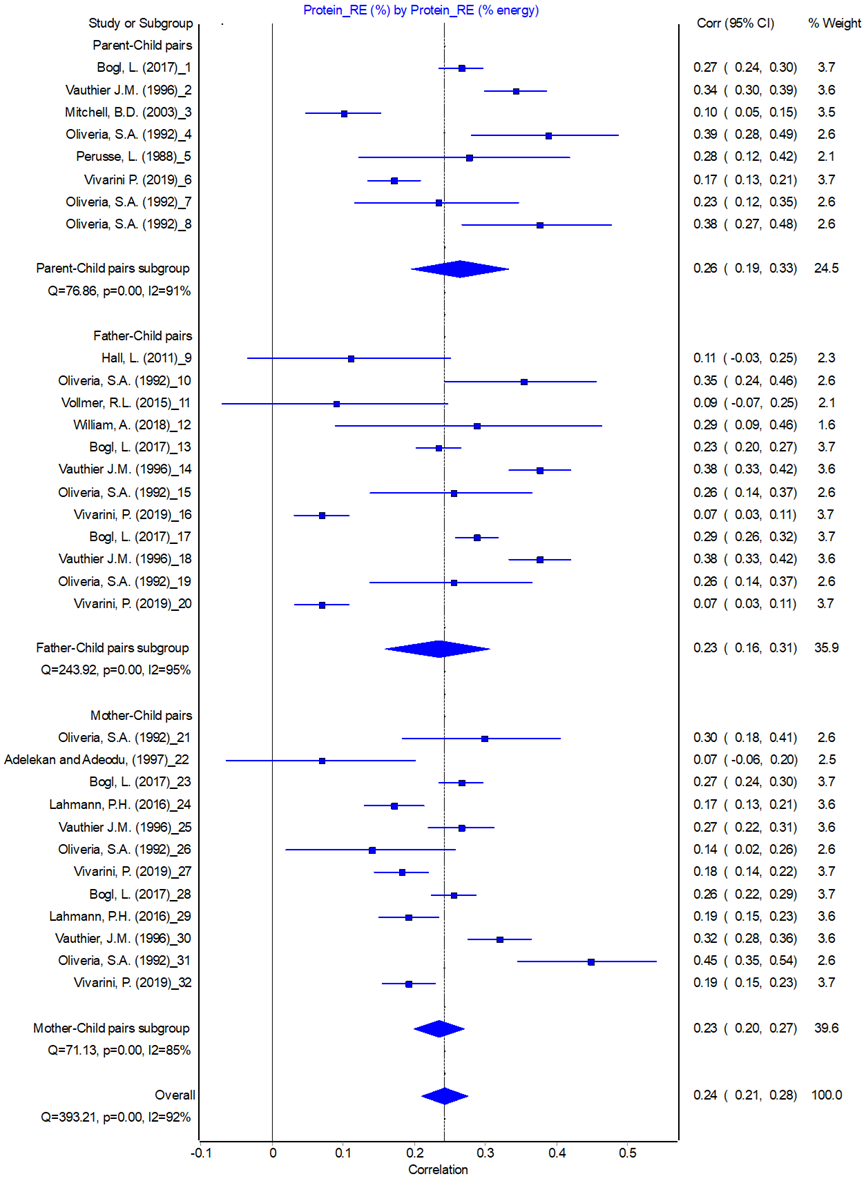
**

**Supplementary Figure 5: Sensitivity analysis of pooled estimation of protein (% energy) intake between parent and child using random effect model.**

**
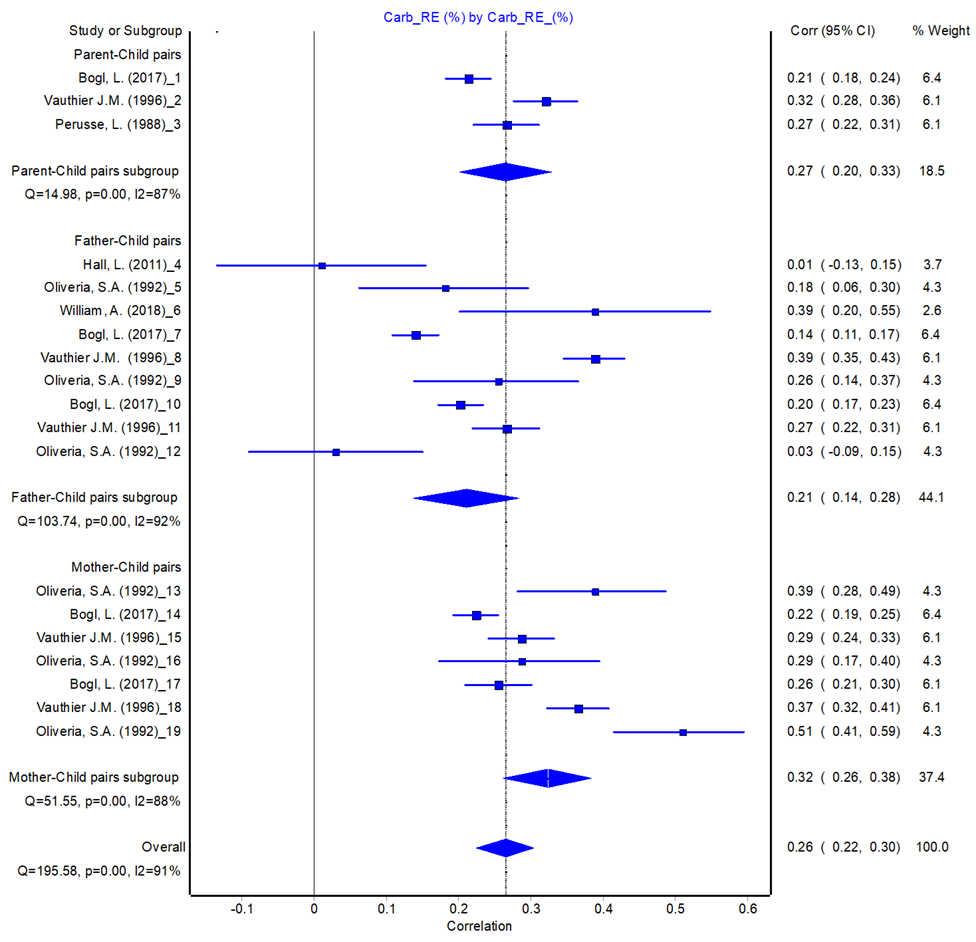
**

**Supplementary Figure 6: Sensitivity analysis of pooled estimation of carbohydrate (% energy) intake between parent and child using random effect model.**

**
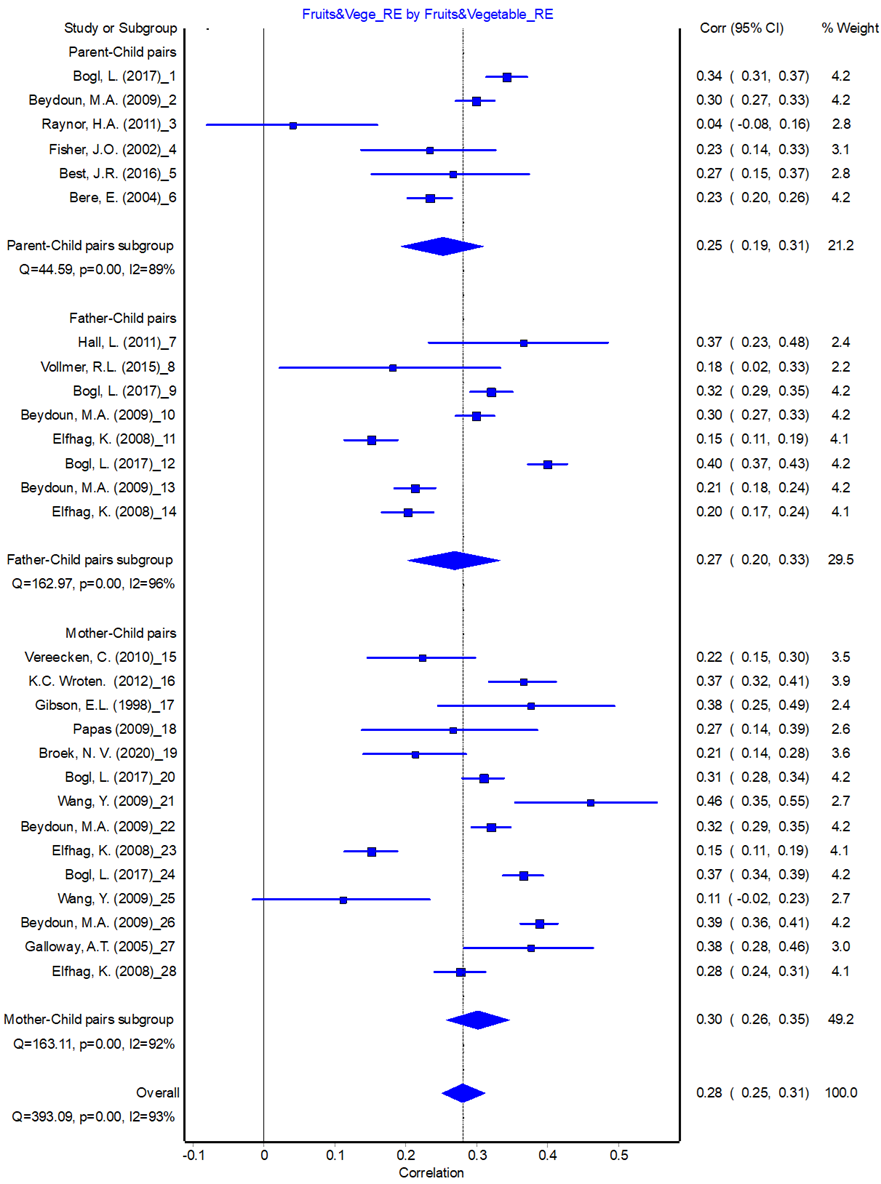
**

**Supplementary Figure 7: Sensitivity analysis of pooled estimation of fruits and vegetables intake between parent and child using random effect model.**

**
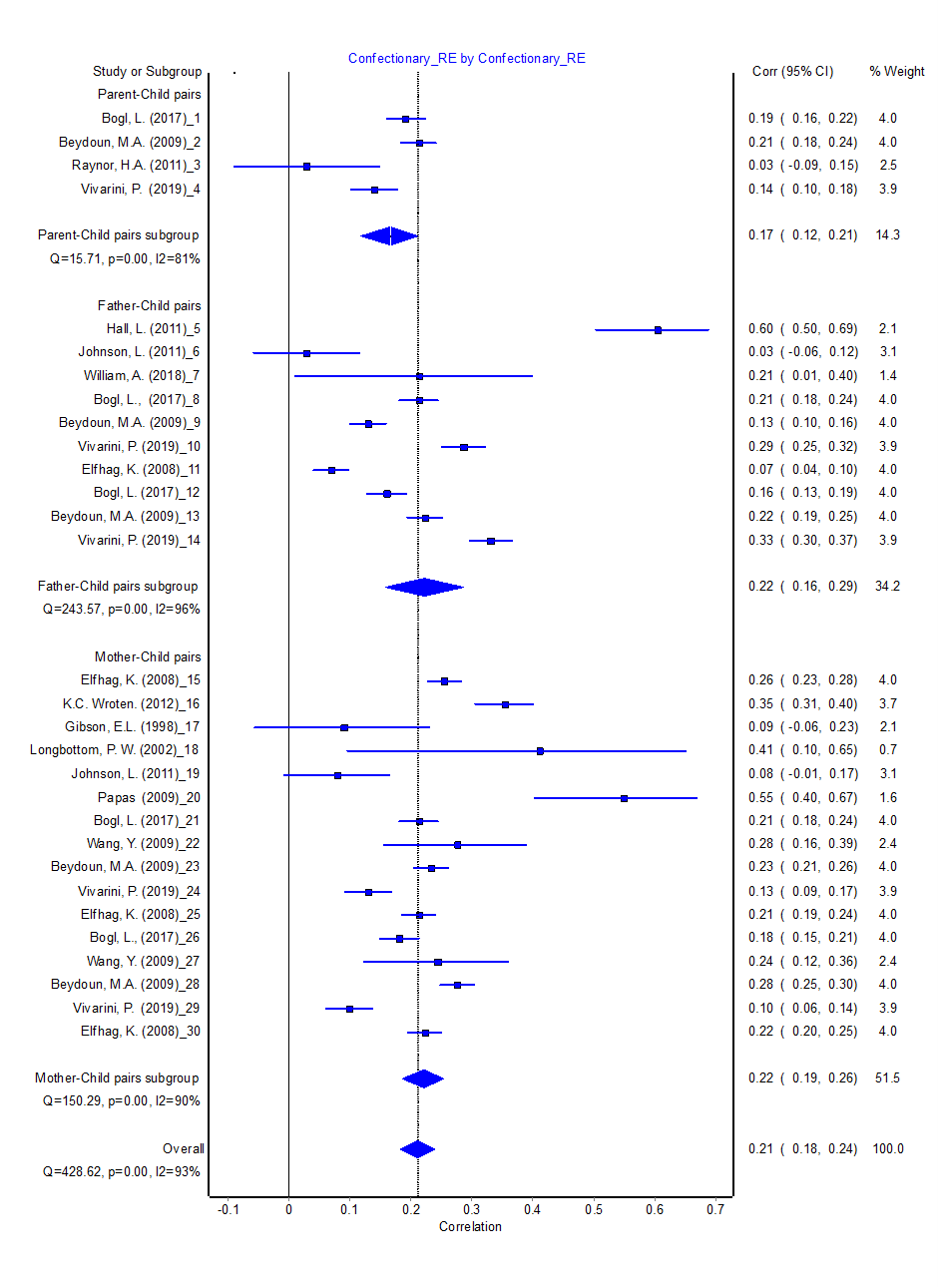
**

**Supplementary Figure 8: Sensitivity analysis of pooled estimation of confectionary foods intake between parent and child using random effect model.**

**
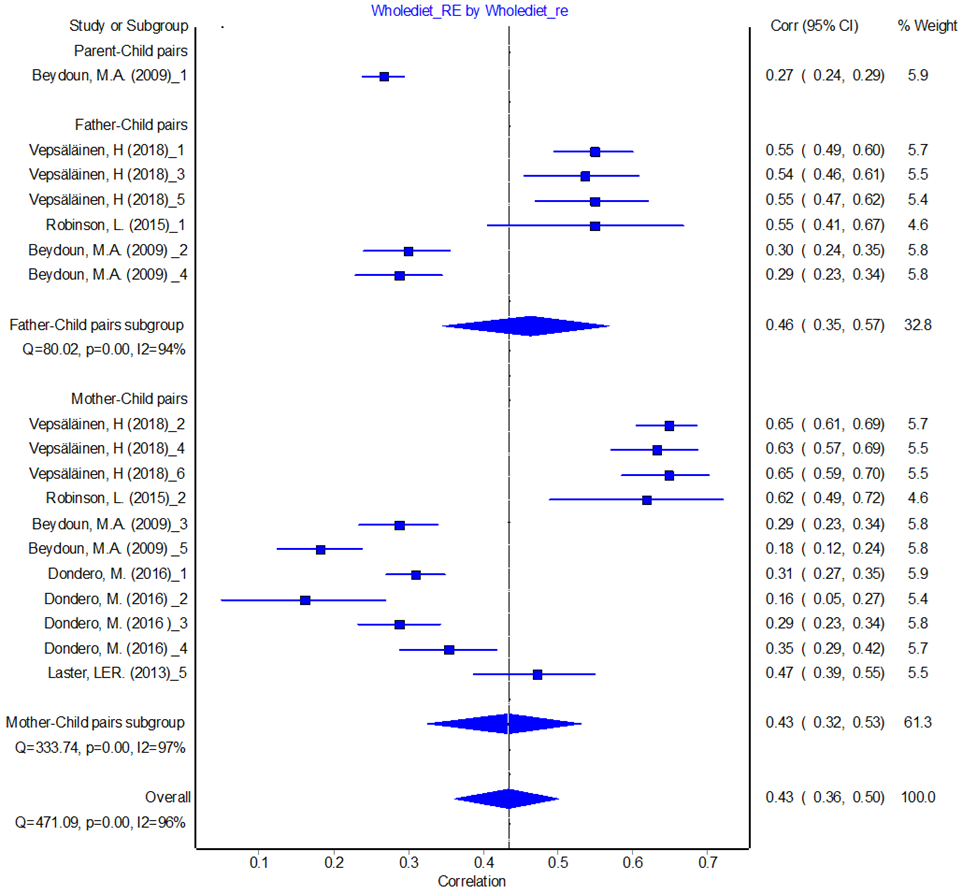
**

**Supplementary Figure 9: Sensitivity analysis of pooled estimation of whole diet between parent and child using random effect model.**

**Table 4: Preferred Reporting Items for Systematic reviews and Meta-Analyses (PRISMA) 2020 checklist**

| **Section/topic** | | **#** | | **Checklist item** | | **Reported on page #** | |
| --- | --- | --- | --- | --- | --- | --- | --- |
| **TITLE** | | | | | |  | |
| Title | | 1 | | Identify the report as a systematic review, meta-analysis, or both. | | 0 | |
| **ABSTRACT** | | | | | |  | |
| Structured summary | | 2 | | Provide a structured summary including, as applicable: background; objectives; data sources; study eligibility criteria, participants; study appraisal and synthesis methods; results; conclusions and implications of key findings; systematic review registration number. | | 3 | |
| **INTRODUCTION** | | | | | |  | |
| Rationale | | 3 | | Describe the rationale for the review in the context of what is already known. | | 4 - 5 | |
| Objectives | | 4 | | Provide an explicit statement of questions being addressed with reference to participants, interventions, comparisons, outcomes, and study design (PICOS). | | 5 | |
| **METHODS** | | | | | |  | |
| Protocol and registration | | 5 | | Indicate if a review protocol exists, if and where it can be accessed (e.g., Web address), and, if available, provide registration information including registration number. | | 5 | |
| Eligibility criteria | | 6 | | Specify study characteristics (e.g., PICOS, length of follow-up) and report characteristics (e.g., years considered, language, publication status) used as criteria for eligibility, giving rationale. | | 5 - 6 | |
| Information sources | | 7 | | Describe all information sources (e.g., databases with dates of coverage, contact with study authors to identify additional studies) in the search and date last searched. | | 5 - 6 | |
| Search | | 8 | | Present full electronic search strategy for at least one database, including any limits used, such that it could be repeated. | | S1 Table | |
| Study selection | | 9 | | State the process for selecting studies (i.e., screening, eligibility, included in systematic review, and, if applicable, included in the meta-analysis). | | 6 | |
| Data collection process | | 10 | | Describe method of data extraction from reports (e.g., piloted forms, independently, in duplicate) and any processes for obtaining and confirming data from investigators. | | 6 | |
| Data items | | 11 | | List and define all variables for which data were sought (e.g., PICOS, funding sources) and any assumptions and simplifications made. | | 6 - 7 | |
| Risk of bias in individual studies | | 12 | | Describe methods used for assessing risk of bias of individual studies (including specification of whether this was done at the study or outcome level), and how this information is to be used in any data synthesis. | | 7 - 8 | |
| Summary measures | | 13 | | State the principal summary measures (e.g., risk ratio, difference in means). | | 7 - 8 | |
| Synthesis of results | | 14 | | Describe the methods of handling data and combining results of studies, if done, including measures of consistency (e.g., I^2^) for each meta-analysis. | | 7 - 8 | |
| **Section/topic** | | **#** | | **Checklist item** | | **Reported on page #** | |
| Risk of bias across studies | | 15 | | Specify any assessment of risk of bias that may affect the cumulative evidence (e.g., publication bias, selective reporting within studies). | | 7 – 8 & S2 Table | |
| Additional analyses | | 16 | | Describe methods of additional analyses (e.g., sensitivity or subgroup analyses, meta-regression), if done, indicating which were pre-specified. | | 7 - 8 | |
| **RESULTS** | | | | | |  | |
| Study selection | | 17 | | Give numbers of studies screened, assessed for eligibility, and included in the review, with reasons for exclusions at each stage, ideally with a flow diagram. | | 8 - 9 | |
| Study characteristics | | 18 | | For each study, present characteristics for which data were extracted (e.g., study size, PICOS, follow-up period) and provide the citations. | | 8 - 9 & S3 Table | |
| Risk of bias within studies | | 19 | | Present data on risk of bias of each study and, if available, any outcome level assessment (see item 12). | | 10 - 11 | |
| Results of individual studies | | 20 | | For all outcomes considered (benefits or harms), present, for each study: (a) simple summary data for each intervention group (b) effect estimates and confidence intervals, ideally with a forest plot. | | 9 - 10 | |
| Synthesis of results | | 21 | | Present results of each meta-analysis done, including confidence intervals and measures of consistency. | | 9 - 10 | |
| Risk of bias across studies | | 22 | | Present results of any assessment of risk of bias across studies (see Item 15). | | 10 - 11 | |
| Additional analysis | | 23 | | Give results of additional analyses, if done (e.g., sensitivity or subgroup analyses, meta-regression [see Item 16]). | | 10 | |
| **DISCUSSION** | | | | | |  | |
| Summary of evidence | | 24 | | Summarize the main findings including the strength of evidence for each main outcome; consider their relevance to key groups (e.g., healthcare providers, users, and policy makers). | | 11 - 13 | |
| Limitations | | 25 | | Discuss limitations at study and outcome level (e.g., risk of bias), and at review-level (e.g., incomplete retrieval of identified research, reporting bias). | | 14 | |
| Conclusions | | 26 | | Provide a general interpretation of the results in the context of other evidence, and implications for future research. | | 14 | |
| **FUNDING** | | | | | |  | |
| Funding | | 27 | | Describe sources of funding for the systematic review and other support (e.g., supply of data); role of funders for the systematic review. | | 9 | |

*From:*  Moher D, Liberati A, Tetzlaff J, Altman DG, The PRISMA Group (2009). Preferred Reporting Items for Systematic Reviews and Meta-Analyses: The PRISMA Statement. PLoS Med 6(7): e1000097. doi:10.1371/journal.pmed1000097

For more information, visit: **www.prisma-statement.org**.

**Reference**

1. Van der Mierden S, Tsaioun K, Bleich A, Leenaars CH. Software tools for literature screening in systematic reviews in biomedical research. *Altex* 2019; **36**(3): 508-17.

2. Vepsalainen H, Nevalainen J, Fogelholm M, et al. Like parent, like child? Dietary resemblance in families. *International Journal of Behavioral Nutrition and Physical Activity* 2018; **15**.

3. Bogl L, Silventoinen K, Hebestreit A, et al. Familial resemblance in dietary intakes of children, adolescents, and parents: Does dietary quality play a role? *Nutrients* 2017; **9**(8): 892.

4. Lahmann PH, Williams GM, Najman JM, Mamun AAJTAjocn. Mother–adult offspring resemblance in dietary intake: a community-based cohort study in Australia. 2016; **105**(1): 185-93.

5. Cullen KW, Lara KM, De Moor C. Familial concordance of dietary fat practices and intake. *Family community health* 2002; **25**(2): 65-75.

6. Robinson LN, Rollo ME, Watson J, Burrows TL, Collins CE. Relationships between dietary intakes of children and their parents: a cross‐sectional, secondary analysis of families participating in the F amily D iet Q uality S tudy. *Journal of Human Nutrition and Dietetics* 2015; **28**(5): 443-51.

7. Wang YF, Caballero B. Resemblance in Dietary Intakes between Urban Low-Income African-American Adolescents and Their Mothers: The Healthy Eating and Active Lifestyles from School to Home for Kids Study. *Journal of the American Dietetic Association* 2009; **109**(1): 52-63.

8. Beydoun M, Wang Y. Parent–child dietary intake resemblance in the United States: evidence from a large representative survey. *J Social science medicine* 2009; **68**(12): 2137-44.

9. Ovaskainen ML, Nevalainen J, Uusitalo L, et al. Some similarities in dietary clusters of pre-school children and their mothers. *British Journal of Nutrition* 2009; **102**(3): 443-52.

10. Feunekes GIJ, Stafleu A, De Graaf C, Van Staveren WA. Family resemblance in fat intake in the Netherlands. *European Journal of Clinical Nutrition* 1997; **51**(12): 793-9.

11. Vereecken C, Haerens L, De Bourdeaudhuij I, et al. The relationship between children's home food environment and dietary patterns in childhood and adolescence. *Public Health Nutrition* 2010; **13**: 1729-35.

12. Billon S, Lluch A, Guéguen R, Berthier AM, Siest G, Herbeth B. Family resemblance in breakfast energy intake: the Stanislas Family Study. *European Journal of Clinical Nutrition* 2002; **56**(10): 1011-9.

13. Stafleu A, Vanstaveren WA, Degraaf C, Burema J, Hautvast JGAJ. Family resemblance in energy, fat, and cholesterol intake: A study among three generations of women. *Preventive Medicine* 1994; **23**(4): 474-80.

14. Wroten KC, O’Neil CE, Stuff JE, Liu Y, Nicklas TA. Resemblance of dietary intakes of snacks, sweets, fruit, and vegetables among mother–child dyads from low income families. *Appetite* 2012; **59**(2): 316-23.

15. Vauthier JM, Lluch A, Lecomte E, Artur Y, Herbeth B. Family resemblance in energy and macronutrient intakes: The Stanislas Family Study. *International Journal of Epidemiology* 1996; **25**(5): 1030-7.

16. Mitchell B, Rainwater D, Hsueh W-C, Kennedy A, Stern M, Maccluer J. Familial aggregation of nutrient intake and physical activity: results from the San Antonio Family Heart Study. *J Annals of epidemiology* 2003; **13**(2): 128-35.

17. Hebestreit A, Intemann T, Siani A, et al. Dietary patterns of European children and their parents in association with family food environment: Results from the i.family study. *Nutrients* 2017; **9**(2).

18. Grimm GC, Harnack L, Story MJJotADA. Factors associated with soft drink consumption in school-aged children. 2004; **104**(8): 1244-9.

19. Gibson EL, Wardle J, Watts CJJA. Fruit and vegetable consumption, nutritional knowledge and beliefs in mothers and children. 1998; **31**(2): 205-28.

20. Hall L, Collins CE, Morgan PJ, Burrows TL, Lubans DR, Callister R. Children's intake of fruit and selected energy-dense nutrient-poor foods is associated with fathers' intake. *Journal of the American Dietetic Association* 2011; **111**(7): 1039-44.

21. Dondero M, Van Hook J. Generational status, neighborhood context, and mother-child resemblance in dietary quality in Mexican-origin families. *Social Science and Medicine* 2016; **150**: 212-20.

22. Fisher JO, Mitchell DC, Smiciklas-Wright H, Birch LL. Maternal milk consumption predicts the tradeoff between milk and soft drinks in young girls' diets. *The Journal of nutrition* 2001; **131**(2): 246-50.

23. Reinaerts E, de Nooijer J, Candel M, de Vries N. Explaining school children's fruit and vegetable consumption: the contributions of availability, accessibility, exposure, parental consumption and habit in addition to psychosocial factors. *Appetite* 2007; **48**(2): 248-58.

24. Oliveria SA, Ellison RC, Moore LL, Gillman MW, Garrahie EJ, Singer MR. Parent-child relationships in nutrient intake: the Framingham Children's Study. *The American journal of clinical nutrition* 1992; **56**(3): 593-8.

25. Raynor HA, Van Walleghen EL, Osterholt KM, et al. The Relationship between Child and Parent Food Hedonics and Parent and Child Food Group Intake in Children with Overweight/Obesity. *Journal of the American Dietetic Association* 2011; **111**(3): 425-30.

26. Cameron AJ, Crawford DA, Salmon J, et al. Clustering of obesity-related risk behaviors in children and their mothers. *Annals of epidemiology* 2011; **21**(2): 95-102.

27. Fisher JO, Mitchell DC, Smiciklas-Wright H, Birch LL. Parental influences on young girls’ fruit and vegetable, micronutrient, and fat intakes. *Journal of the American dietetic association* 2002; **102**(1): 58-64.

28. Longbottom P, Wrieden W, Pine C. Is there a relationship between the food intakes of Scottish 5½− 8½‐year‐olds and those of their mothers? *Journal of Human Nutrition and Dietetics* 2002; **15**(4): 271-9.

29. Best J, Goldschmidt A, Mockus-Valenzuela D, Stein R, Epstein L, Wilfley D. Shared weight and dietary changes in parent–child dyads following family-based obesity treatment. *Health Psychology* 2016; **35**(1): 92.

30. Perusse L, Tremblay A, Leblanc C, et al. Familial resemblance in energy intake: contribution of genetic and environmental factors. *The American journal of clinical nutrition* 1988; **47**(4): 629-35.

31. Vollmer RL, Adamsons K, Gorin A, Foster JS, Mobley AR, Dietetics. Investigating the relationship of body mass index, diet quality, and physical activity level between fathers and their preschool-aged children. *Journal of the Academy of Nutrition and Dietetics* 2015; **115**(6): 919-26.

32. Galloway AT, Fiorito L, Lee Y, Birch LL. Parental pressure, dietary patterns, and weight status among girls who are “picky eaters”. *Journal of the American Dietetic Association* 2005; **105**(4): 541-8.

33. Laskarzewski P, Morrison JA, Khoury P, et al. Parent-child nutrient intake interrelationships in school children ages 6 to 19: the Princeton School District Study. *The American journal of clinical nutrition* 1980; **33**(11): 2350-5.

34. Johnson L, Van Jaarsveld CH, Wardle J. Individual and family environment correlates differ for consumption of core and non-core foods in children. *British Journal of Nutrition* 2011; **105**(6): 950-9.

35. Hannon PA, Bowen DJ, Moinpour CM, McLerran DF. Correlations in perceived food use between the family food preparer and their spouses and children. *Appetite* 2003; **40**(1): 77-83.

36. Murrin CM, Heinen MM, Kelleher CC. Are Dietary Patterns of Mothers during Pregnancy Related to Children's Weight Status? Evidence from the Lifeways Cross-Generational Cohort Study. *AIMS public health* 2015; **2**(3): 274.

37. Lipsky LM, Haynie DL, Liu A, Nansel TR. Resemblance of Diet Quality in Families of Youth with Type 1 Diabetes Participating in a Randomized Controlled Behavioral Nutrition Intervention Trial in Boston, MA (2010-2013): A Secondary Data Analysis. *Journal of the Academy of Nutrition and Dietetics* 2019; **119**(1): 98-105.

38. Stanton CA, Fries EA, Danish SJ. Racial and gender differences in the diets of rural youth and their mothers. *American journal of health behavior* 2003; **27**(4): 336-47.

39. Patterson TL, Rupp JW, Sallis JF, Atkins CJ, Nader PR. Aggregation of dietary calories, fats, and sodium in Mexican-American and Anglo families. *American journal of preventive medicine* 1988; **4**(2): 75-82.

40. Vivarini P, Kerr JA, Clifford SA, et al. Food choices: concordance in Australian children aged 11–12 years and their parents. *BMJ open* 2019; **9**(Suppl 3): 147-56.

41. Elfhag K, Tholin S, Rasmussen F. Consumption of fruit, vegetables, sweets and soft drinks are associated with psychological dimensions of eating behaviour in parents and their 12-year-old children. *Public health nutrition* 2008; **11**(9): 914-23.

42. Bere E, Klepp K-I. Correlates of fruit and vegetable intake among Norwegian schoolchildren: parental and self-reports. *Public health nutrition* 2004; **7**(8): 991-8.

43. Rossow I, Rise J. Concordance of parental and adolescent health behaviors. *Social science & medicine* 1994; **38**(9): 1299-305.

44. Adelekan D, Adeodu O. Interrelationship in nutrient intake of Nigerian mothers and their children: nutritional and health implications. *African journal of medicine and medical sciences* 1997; **26**(1-2): 63-5.

45. Feunekes GIJ, de Graaf C, Meyboom S, van Staveren WA. Food choice and fat intake of adolescents and adults: associations of intakes within social networks. *Preventive Medicine* 1998; **27**(5 part 1): 645-56.

46. Runyan S, Stadler D, Bainbridge C, Miller S, Moyer-Mileur L. Familial resemblance of bone mineralization, calcium intake, and physical activity in early-adolescent daughters, their mothers, and maternal grandmothers. *Journal of the American Dietetic Association* 2003; **103**(10): 1320-5.

47. Park H, Yim K, Cho S-I. Gender differences in familial aggregation of obesity-related phenotypes and dietary intake patterns in Korean families. *Annals of epidemiology* 2004; **14**(7): 486-91.

48. da Veiga G, Sichieri R. Correlation in food intake between parents and adolescents depends on socioeconomic level. *Nutrition Research* 2006; **26**(10): 517-23.

49. López-Alvarenga JC, Vázquez-Velázquez V, Bolado-García VE, et al. Influencia de los padres sobre las preferencias alimentarias en niños de dos escuelas primarias con diferente estrato económico. Estudio ESFUERSO. *Gaceta Médica de México* 2007; **143**(6): 463-9.

50. Papas MA, Hurley KM, Quigg AM, Oberlander SE, Black MM. Low-income, African American adolescent mothers and their toddlers exhibit similar dietary variety patterns. *Journal of Nutrition Education & Behavior* 2009; **41**(2): 87-94.

51. Rozin P. Family resemblance in food and other domains: The family paradox and the role of parental congruence. *Appetite* 1991; **16**(2): 93-102.

52. Hart CN, Raynor HA, Jelalian E, Drotar D. The association of maternal food intake and infants' and toddlers' food intake. *Child: Care, Health and Development* 2010; **36**(3): 396-403.

53. Kunaratnam K, Halaki M, Wen LM, Baur LA, Flood VM. Mother-child dietary behaviours and their observed associations with socio-demographic factors: findings from the Healthy Beginnings Trial. *British Journal of Nutrition* 2018; **119**(4): 464-71.

54. Prichard I, Hodder K, Hutchinson A, Wilson C. Predictors of mother-daughter resemblance in dietary intake. The role of eating styles, mothers' consumption, and closeness. *Appetite* 2012; **58**(1): 271-6.

55. Tada Y, Tomata Y, Sunami A, et al. Examining the relationship between vegetable intake of mothers and that of their children: a cross-sectional study of 10- to 12-year-old schoolchildren in Japan. *Public Health Nutrition* 2015; **18**(17): 3166-71.

56. Broek NV, Larsen J, Verhagen M, Burk W, Vink J. Is Adolescents Food Intake Associated with Exposure to the Food Intake of Their Mothers and Best Friends? *Nutrients* 2020; **12**.

57. Thorsdottir I, Gunnarsdottir I, Ingolfsdottir S, Palsson G. Fruit and vegetable intake: vitamin C and β-carotene intake and serum concentrations in six-year-old children and their parents. *Scandinavian Journal of Food and Nutrition* 2006; **50**(2): 71-6.

58. Laster L, Lovelady C, West D, et al. Diet quality of overweight and obese mothers and their preschool children. *Journal of the Academy of Nutrition and Dietetics* 2013; **113**(11): 1476-83.

59. Vanhala M, Laitinen J, Kaikkonen K, Keinänen‐Kiukaanniemi S, Korpelainen R. Parental predictors of fruit and vegetable consumption in treatment‐seeking overweight children. *Journal of Human Nutrition and Dietetics* 2011; **24**(1): 47-53.

60. Harris T, Ramsey M. Paternal modeling, household availability, and paternal intake as predictors of fruit, vegetable, and sweetened beverage consumption among African American children. *Appetite* 2015; **85**: 171-7.

61. Williams A, de Vlieger N, Young M, et al. Dietary outcomes of overweight fathers and their children in the Healthy Dads, Healthy Kids community randomised controlled trial. *Journal of Human Nutrition and Dietetics* 2018; **31**(4): 523-32.

62. Zuercher JL, Wagstaff DA, Kranz S. Associations of food group and nutrient intake, diet quality, and meal sizes between adults and children in the same household: a cross-sectional analysis of U.S. households. *Nutrition Journal* 2011; **10**: 131-45.

63. Hosseini-Esfahani F, Zahedi AS, Akbarzadeh M, et al. The resemblance of dietary intakes in three generations of parent-offspring pairs: Tehran lipid and glucose study. *Appetite* 2022; **169**: 105794.

64. Mirmiran P, Zahedi AS, Koochakpour G, et al. Resemblance of nutrient intakes in three generations of parent-offspring pairs: Tehran lipid and Glucose Study. *PLoS One* 2022; **17**(4): e0266941.
